# Construction of sRNA Regulatory Network for *Magnaporthe oryzae* Infecting Rice Based on Multi-Omics Data

**DOI:** 10.3389/fgene.2021.763915

**Published:** 2021-11-12

**Authors:** Enshuang Zhao, Hao Zhang, Xueqing Li, Tianheng Zhao, Hengyi Zhao

**Affiliations:** ^1^ College of Software, Jilin University, Changchun, China; ^2^ College of Computer Science and Technology, Jilin University, Changchun, China

**Keywords:** *Magnaporthe oryzae*, rice, multi-omics, sRNA, protein, machine learning

## Abstract

Studies have shown that fungi cause plant diseases through cross-species RNA interference mechanism (RNAi) and secreted protein infection mechanism. The small RNAs (sRNAs) of *Magnaporthe oryzae* use the RNAi mechanism of rice to realize the infection process, and different effector proteins can increase the autotoxicity by inhibiting pathogen-associated molecular patterns triggered immunity (PTI) to achieve the purpose of infection. However, the coordination of sRNAs and proteins in the process of *M. oryzae* infecting rice is still poorly understood. Therefore, the combination of transcriptomics and proteomics to study the mechanism of *M. oryzae* infecting rice has important theoretical significance and practical value for controlling rice diseases and improving rice yields. In this paper, we used the high-throughput data of various omics before and after the *M. oryzae* infecting rice to screen differentially expressed genes and sRNAs and predict protein interaction pairs based on the interolog and the domain-domain methods. We were then used to construct a prediction model of the *M. oryzae*-rice interaction proteins according to the obtained proteins in the proteomic network. Finally, for the differentially expressed genes, differentially expressed sRNAs, the corresponding mRNAs of rice and *M. oryzae*, and the interacting protein molecules, the *M. oryzae*-rice sRNA regulatory network was built and analyzed, the core nodes were selected. The functional enrichment analysis was conducted to explore the potential effect pathways and the critical infection factors of *M. oryzae* sRNAs and proteins were mined and analyzed. The results showed that 22 sRNAs of *M. oryzae*, 77 secretory proteins of *M. oryzae* were used as effect factors to participate in the infection process of *M. oryzae*. And many significantly enriched GO modules were discovered, which were related to the infection mechanism of *M. oryzae*.

## Introduction

Rice is an important crop, providing a portion of staple food for more than half of the world’s population (Ruiz-Sánchez et al., 2010). However, rice blast is the most severe disease of rice, caused by *Magnaporthe oryzae*, which seriously affects crop stability and sustainability around the world (Imam et al., 2015). Therefore, research on how to control rice blast is widespread.

Although *M. oryzae* is a model fungus for the study of plant-fungal diseases, current studies have shown that the long-term control performance of rice blast by using rice fungicides in the field or selecting rice varieties resistant to *M. oryzae* is still unstable (Deng and Naqvi, 2019). Therefore, people have done a lot of research on *M. oryzae* infecting rice and achieved some research results. However, the interaction mechanism between fungi and plants is very complicated, and it is currently challenging to analyze the molecular interaction mechanism only by biological experiments (Li et al., 2017; Nelson et al., 2018). Therefore, researchers began to use biocomputing methods to assist and guide biological experiments based on the emergence of many omics data related to fungus-plant interactions, such as genomics, transcriptomics, proteomics and metabolomics multi-omics data to reveal interactions between biomolecules and explore key factors in biological processes.

For exploring the key biomolecules in the process of fungus-plant interactions small RNAs (sRNAs) were first studied in depth. sRNAs refer to those that do not encode proteins in the organism and are mostly 18nt-40nt in length (Mueth et al., 2015). The common mechanism of action of sRNAs is RNA interference (RNAi). The effector complex RISC is added to one of the sRNA strands to achieve the purpose of inhibiting protein biosynthesis (Majumdar et al., 2017). Researchers have found that using the host plant’s RNAi mechanism by pathogenic sRNAs to achieve the infection process may be ubiquitous in the fungus-plant interaction mechanism (Weiberg et al., 2013; Cai et al., 2018).

In addition, fungi as eukaryotes, their secreted proteins are transported across the membrane by endocytosis and exocytosis (Pompa et al., 2017; Riquelme et al., 2018). Secreted proteins are proteins produced by the nucleus, processed and transported through the endoplasmic reticulum and Golgi apparatus, and secreted outside of cells or other cells. They play key biological regulatory roles, such as hormones, antibodies, and enzymes (Faso et al., 2009). In addition, studies have found that pathogens invade hosts through secreted proteins to achieve an attack on the hosts’ immune effect. For example, when soil-borne pathogenic fungi invade plants, they secrete an effector protein (Verticillium dahliae polysaccharide deacetylase, VdPDA1), which deacetylates chitin oligosaccharides produced by plants to resist infection by pathogens, thus reducing or inactivating the immune system of plants, to achieve the purpose of infection (Cui et al., 2020).

However, at present, the research on the mechanism of fungus-plant interaction is still in its infancy (Kim et al., 2016; Larsen et al., 2016; Großkinsky et al., 2018; Wang et al., 2019). In addition to genomics research combining plant disease resistance genes and sRNA for analysis (Zhang et al., 2016; Raman et al., 2017), other omics analysis is still based on single omics analysis, and some sRNAs (Zhang et al., 2019; Chang et al., 2020), proteins (Solomon and Oliver, 2001; Grenville-Briggs et al., 2005; Grohmann and Bronte, 2010; McGaha et al., 2012; Yang et al., 2012), metabolites (Parker et al., 2009) have been identified. In fungi infecting plants, how sRNA and protein molecules are involved in the regulation is still unknown. Therefore, based on differentially expressed genes, differentially expressed sRNAs and protein interaction pairs in the process of *M. oryzae* infecting rice, this study proposed a new method to analyze the multi-omics data of *M. oryzae* infecting rice and constructed a multi-omics data integration-based *M. oryzae*-rice interaction network. It also wholly presented the interaction relationship between the markers of various omics in the process of *M. oryzae* infecting rice and revealed the key nodes that play a regulatory role in *M. oryzae* infection in rice. This paper found a possible solution for studying the mechanism of *M. oryzae* infecting rice and provided research ideas for preventing and controlling rice and other food crops.

## Data and Methods

**FIGURE 1:**
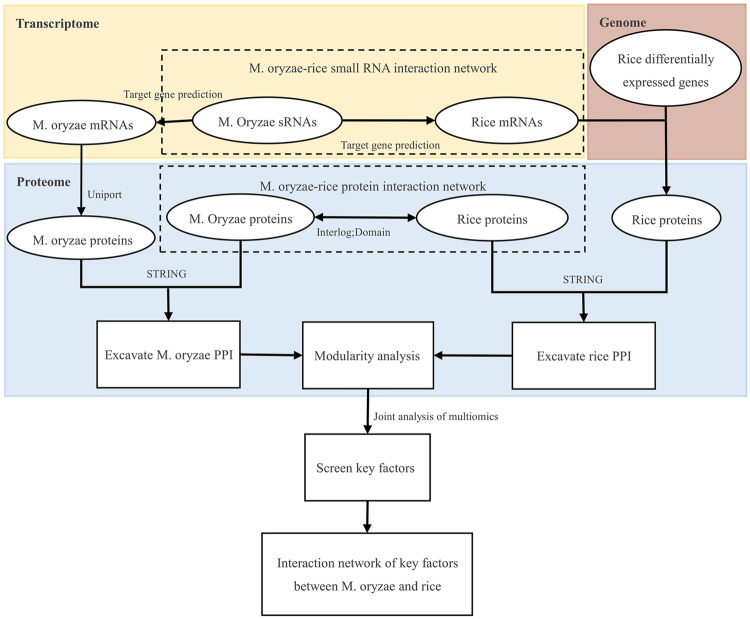
Overall design route.

Firstly, the genomic, transcriptome, and proteome data were analyzed to establish the *M. oryzae*-rice sRNA interaction network and *M. oryzae*-rice protein interaction network. Then, the sRNA and protein interaction networks of *M. oryzae* and rice were analyzed. Finally, the PPI interaction networks and GO functional enrichment modules of *M. oryzae* and rice were excavated, respectively, and the key factors of multiple omics joint regulations and the biological processes involved were explored. The design roadmap for this work is shown in Figure 1.

### Data Source

Regarding the genome and transcriptome, this paper used the gene chip expression data of rice before and after *M. oryzae* infection with rice at 72 h, sRNA data of *M. oryzae* cultured on a complete medium for 16 h, the mixed sRNA data of the rice infected by *M. oryzae* for 72 h (Raman et al., 2013), the gene expression data of rice after 48 h of culture, the gene expression data of rice after 48 h of infection by *M. oryzae* (Chujo et al., 2013), and the mRNA data of rice. These are all from the NCBI database. Regarding the proteome, high-throughput protein data of mode hosts, mode pathogens, rice and *M. oryzae* were obtained from HPIDB, NCBI and Uniport databases. We first obtained the protein IDs of *M. oryzae* and rice from the NCBI and Uniport databases. Because different databases have different identifiers for the same protein, the obtained protein IDs must be converted uniformly. Here, the protein IDs of the Uniport database were selected as the unified protein ID identifiers, and the high-throughput data of these proteins were obtained after the protein IDs were converted.

### Data Preprocessing

#### The Acquisition of Differentially Expressed Genes in Rice

The commonly used R software packages for the gene chip probe level data processing include affy, affyPLM, affycomp, gcrma, etc. In this step, the affy package was used to analyze the rice gene differential expression. Firstly, the background noise of the gene chips was denoised by the MAS method. Then, in order to eliminate the influence of signal strength and other factors between different chips, the linear normalization method was used for chip data. Next, the expression amount of the gene probes was calculated by the hybridization signal of the probeset using the function computeExprSet in the affy package.

**FIGURE 2:**
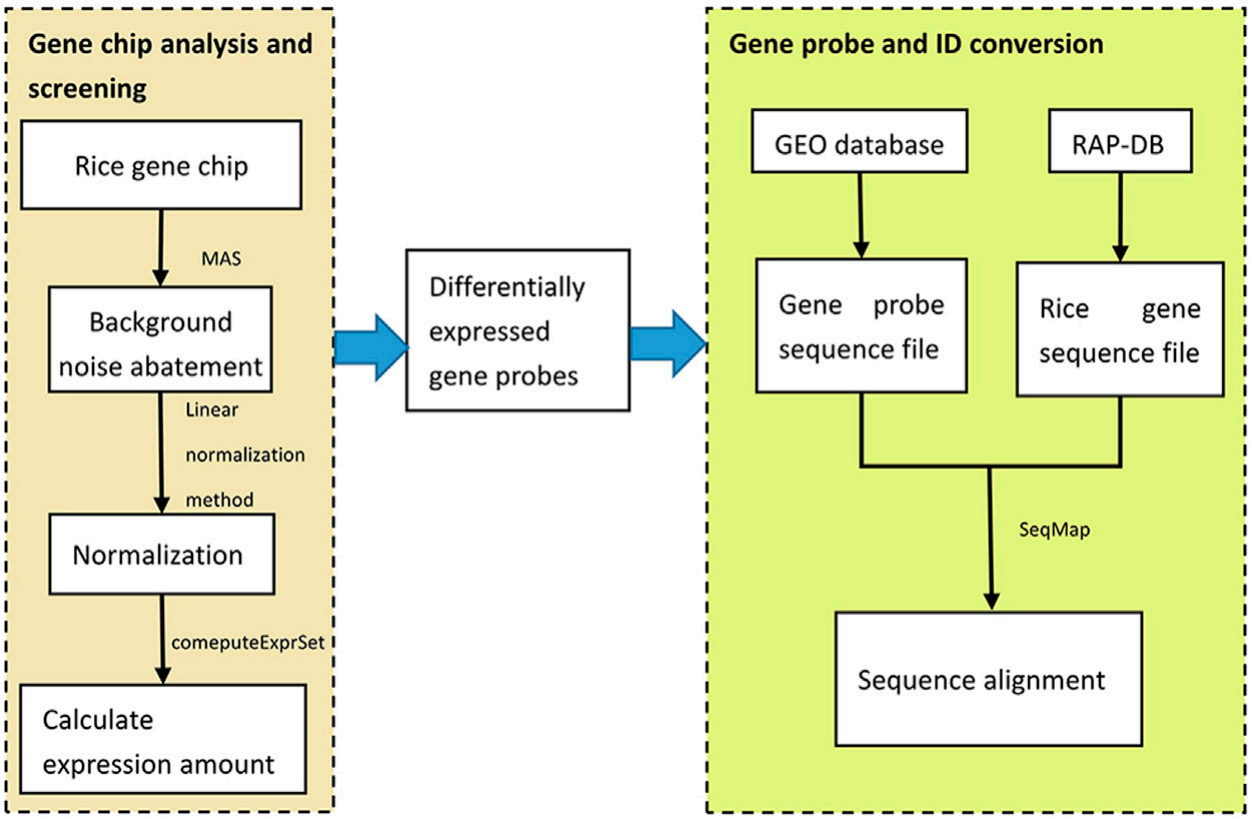
Algorithms for the analysis of differentially expressed genes.

Then, the sequence number of the probes used by the gene chip was retrieved from the GEO database and the probe sequences were downloaded. Then, the whole rice genome sequences were downloaded from RAP-DB, and the sequence alignment between the gene probe sequences and the whole rice genome sequences was performed by using the SeqMap sequence alignment tool to find the rice gene IDs corresponding to the gene probes. Finally, by extracting the matched rice gene IDs, the conversion from gene probes to gene IDs was completed, and 1.5-fold differentially expressed rice genes were screened out, totaling 1,368. This process is shown in Figure 2.

#### Differentially Expressed sRNAs Screening of *M. oryzae*

First, to remove the adapters and get the correct sRNA sequences, the cutadapt tool was used to remove the sRNA data adapters. Next, genome matching was performed on the sRNA data of *M. oryzae* after removing the adapters to remove the sRNA data that were not of *M. oryzae* from the data. The specific operation was to perform the mapping operation on the mixed sRNA data of *M. oryzae* after removing the adapters and match it to the genome of *M. oryzae* to obtain the pure sRNA data of *M. oryzae*. The genome matching tools used in this section were bowtie and samtools.

Since there are several sRNA sequences of different lengths in FASTQ files, it is necessary to control the length of these sRNA sequences. According to the available length of plant sRNAs, we selected the sRNA sequences of *M. oryzae* from 18nt to 25nt, and suggested that these sRNAs could be used to predict the target genes of rice. File A containing sRNA sequence, sequence length and sequence expression amount of *M. oryzae* was obtained from the *M. oryzae* sRNA data after length control and without genome mapping. Then, the file after genome mapping was extracted, and each sRNA sequence of *M. oryzae* was extracted into file B. Finally, the two files were matched. After matching each sRNA sequence in file A, *M. oryzae* sRNA data appearing in file B was output.

In this paper, the 3/4 quantile normalization method was used to normalize the sRNA expression amount data before and after the infection of *M. oryzae*. The specific method was to rank the sRNA expression amount of *M. oryzae* from high to low and find the *M. oryzae* sRNA ranked in 3/4. Then, this expression amount was taken as the baseline of the lower expression level, and the expression amounts of other samples were converted to multiples of this expression amount. Finally, the data of *M. oryzae* differentially expressed sRNA after normalized treatment were statistically analyzed, and the expression amount and expression rate was used for screening. The following formula calculated the expression rate:

$$Growth\_Rate=\frac{count_{after}-count_{before}}{count_{before}}$$

**FIGURE 3:**
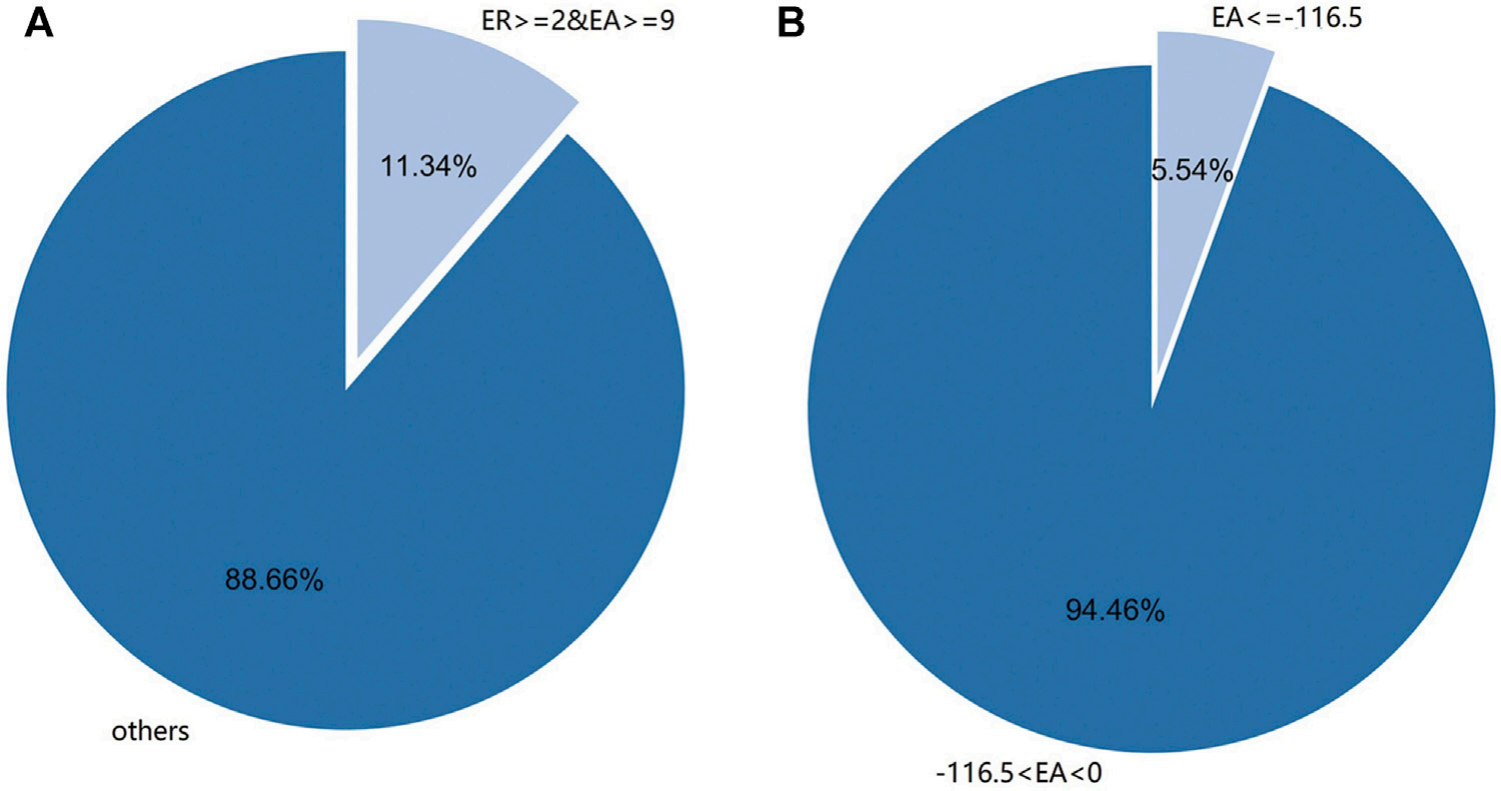
**(A)** The distribution of differential expression rate (ER) of sRNAs of *M. oryzae* was greater than or equal to 2 and the expression amount (EA) was more significant than or equal to 9. **(B)** The distribution of differential EA of sRNAs of *M. oryzae* was less than or equal to −116.5.

It was found that there were 4933 new sRNA data after infection, and the expression amount was sorted, and the top 146 sRNA data with the highest expression amount were selected. The data of 6,100 sRNA species before and after the infection of *M. oryzae* were screened by two conditions: expression amount and expression rate. A total of 220 species *M. oryzae* sRNAs were screened out by selecting sRNAs whose differential expression amount increment was more significant than or equal to 9 and differential expression increase rate was more significant than or equal to 2. Similarly, the sRNA data of *M. oryzae* with differential expression amount increment less than −116.5 were selected, and there were 257 kinds of sRNA data. The differential expression amount increment and expression amount increase rate of sRNAs above were all greater than the corresponding mean values of increase or decrease. Because the sRNA differential expression increase rate ranged from 0 to 1, and the change rate was meager, only the increment of differential expression amount was used to screen the decreased expression sRNA of *M. oryzae*. The distribution map of *M. oryzae* differentially expressed sRNAs is shown in Figure 3.

#### Preprocessing of Protein Data

Blast sequence alignment was performed on the protein amino acid sequences of downloaded rice and *M. oryzae*. Proteins with sequence similarity more significant than 95 were removed as repeated proteins to eliminate the error in the same protein sequencing by different sequencing platforms and avoid duplicating the same protein that was considered to be caused by two different proteins.

### Prediction of *M. oryzae*-Rice sRNA Interaction Pairs

Using the bioinformatics method to accurately and rapidly predict the target genes of miRNA can provide clues for studying the function of miRNA. Using target gene prediction software to predict miRNA target genes is more efficient and faster than experimental biological methods. There are many standard target gene tools, including TargetScan, miRcode, miRDB, RNA22, and tapir, the target gene prediction tool used in this paper. Before the prediction, T was converted to U in the sRNA data and such sequence files were converted to FASTA files. After the sequence base conversion of FASTA files, the tapir tool can be used to predict the target genes of the sRNA sequence files of *M. oryzae*.

First, the FASTA CDS files of *M. oryzae* and rice were downloaded, and the FASTA files of the differentially expressed *M. oryzae* sRNAs were obtained. Then, when the tapir tool was used for target gene prediction, the matching score was set as 0.5 and the free energy ratio was set as 0.7. After target gene matching, Python script was applied to process the prediction results, and the final target gene prediction result file was obtained.

In this section, 366 kinds of differential expression amount up-regulated and newly added of *M. oryzae* sRNAs were targeted to rice mRNAs. A total of 1,857 rice mRNAs were obtained. After gene IDs matching and deduplication of these mRNAs, 1,121 rice gene IDs were obtained. In the same way, 257 kinds of *M. oryzae* sRNAs with down-regulated differential expression amounts were targeted to *M. oryzae*, and 664 *M. oryzae* mRNAs and 264 *M. oryzae* genes were obtained.

### Prediction of *M. oryzae*-Rice Protein Interaction Pairs

#### Sequence-Based

**FIGURE 4:**
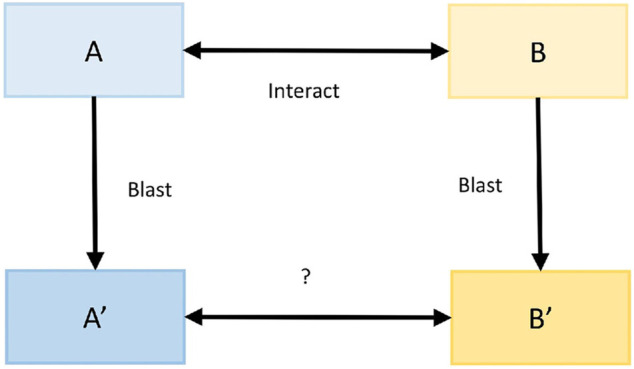
Interolog method.

The protein interaction prediction method based on sequence features (interolog method) is based on the principle that homologous proteins have similar functional and structural characteristics (Thanasomboon et al., 2017). Here, the interspecific interolog method predicted the protein interaction relationship between *M. oryzae* and rice. First, the confirmed interaction mode host and mode pathogen protein sequences were recorded as A and B, while the protein sequences of rice and *M. oryzae* were recorded as A′and B′. Then, for each protein amino acid sequence in A′, sequence alignment was carried out with the protein amino acid sequences in A, and the accuracy was obtained. Similarly, file B also followed this step. Finally, the accuracy of the interaction relationship pairs between A′and B′ was calculated by interacting with the proteins in A and B. The process is shown in Figure 4.

In this process, the interolog method was used to screen the protein interaction pairs between rice and *M. oryzae*, and the threshold was set as E-value less than or equal to 1E-5 and similarity greater than or equal to 30. Then, the model pathogen and mode host protein pairs corresponding to *M. oryzae* and rice proteins were matched, and the protein pair files of *M. oryzae* and rice were obtained based on the interolog method.

#### Domain-Based

**FIGURE 5:**
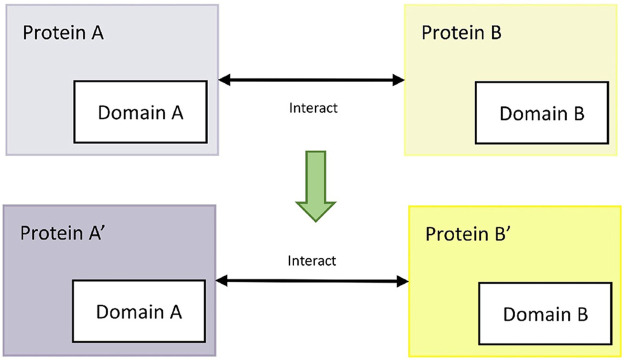
Domain-domain method.

The available domain-based protein interaction prediction method (domain-domain interaction method) is based on the principle that interacting protein pairs may have the exact functional domains (Lee et al., 2006). For example, for the confirmed interactions between mode host protein A and mode pathogen protein B, if rice protein A′and *M. oryzae* protein B′ have the same interaction functional domains as protein A and protein B, then rice protein A′and *M. oryzae* protein B′ interact. The process is shown in Figure 5.

In this process, functional domains were obtained from the protein amino acid sequences of mode hosts, mode pathogens, rice and *M. oryzae* through the Pfam database. E-value was selected as 1E-5 and the coincidence rate was selected as 90%. TSV files containing protein IDs, protein functional domains and E-values were obtained. Then, protein domain files were extracted and sorted to obtain protein interaction relationship pairs based on functional domains.

#### Prediction of Secreted Protein of *M. oryzae*

By combining the interolog method and domain-domain method, 83664 pairs of protein interactions were obtained following the two methods. However, not all *M. oryzae* proteins can be transported across the membrane, it is necessary to do the secreted protein identification of the above *M. oryzae* proteins and screen out the *M. oryzae*-rice protein interaction network that *M. oryzae* proteins were secreted proteins.

In this paper, the secreted proteins of *M. oryzae* were predicted on TMHMM. The FASTA files of 323 *M. oryzae* protein amino acid sequences were obtained through the Uniport database and imported into the TMHMM website to obtain their secreted proteins’ predicted results. When the expected number of amino acids in the transmembrane helix of a protein is greater than or equal to 18, or when the transmembrane helix number of N-the best predicted is greater than or equal to 1, the protein can be considered a secreted protein. Therefore, protein IDs with parameters ExpAA greater than or equal to 18 or PredHel greater than or equal to 1 were extracted. The obtained *M. oryzae* secreted proteins were matched and screened with the previous 83664 *M. oryzae*-rice protein interaction pairs, and finally, 7352 *M. oryzae*-rice protein interaction pairs were obtained.

### Construction of a Prediction Model for Cross-Species Regulatory Protein Pairs Between *M. oryzae* and Rice

#### Acquisition and Processing of Positive and Negative Samples

This paper established a prediction model for *M. oryzae* and rice interaction protein pairs and obtained protein interaction pairs through the sequence and functional structure prediction in experiments. The 7352 data of the effective interaction pairs of *M. oryzae* and rice obtained above were used as positive samples. The negative samples were randomly selected from other *M. oryzae* rice protein interaction pairs except the positive samples that the ratio of positive and negative samples was 1:1.

For the protein features of *M. oryzae* and rice, the proteins’ amino acid sequences and functional domains were used as the feature data. In addition, functional domain texts were preprocessed before training, including unifying special symbols, spaces, upper and lower case letters of each functional domain and removing stop words to achieve standardized processing of data samples.

#### Construction of Protein Interaction Pair Prediction Model Based on textRNN

Recurrent Neural Network (RNN) is mainly used in sequence prediction, character generation, emotion recognition, man-machine dialogue, etc. RNN is a kind of recursive neural network that takes sequence data as input, recurses in the sequence’s evolution direction, and connects all nodes in a chain. The sequence information determines the task of the event itself, which requires previous knowledge and current information to determine the output result jointly. As a result, textRNN can more effectively address the problem of contextual semantic relevance. Considering that the protein’s amino acid sequence and functional domains belonged to short texts, which have contextual semantic relevance characteristics, this paper used textRNN to construct the protein interaction pair binary classification model.

A multi-layer RNN network needs to be established in the construction of RNN model. The dropout layer was added after each RNN kernel function, and the amino acid sequences after the *M. oryzae* and rice protein interaction pair segmentation were used as the input variable of the RNN model. The first hidden layer activated this input. Then the successive activations were performed layer by layer to get the output. Each hidden layer had its own weight and bias. Parameters such as the classification results, accuracy and loss function of the output protein interaction pairs were output by the output layer. The optimal RNN protein interaction model was obtained by adjusting learning_rate, dropout_keep_prob and total iteration cycles according to the learning curve and confusion matrix. Finally, different evaluation indexes were applied to evaluate and verify the model. The accuracy of protein interaction pairs predicted by the interolog method and domain-domain method in this paper was proved.

### Analysis of Regulatory Network Between *M. oryzae* and Rice

In order to analyze the obtained sRNA and protein interaction network of *M. oryzae*-rice, and the network diagram of *M. oryzae*-rice protein interaction was too significant. Therefore, the PPI networks of *M. oryzae* and rice jointly regulated by various omics were explored, respectively. First, the PPI network of *M. oryzae* was mined based on the proteins regulated by *M. oryzae* differentially expressed sRNAs and *M. oryzae* proteins in the *M. oryzae*-rice protein interaction network. And the PPI network of rice was mined based on the proteins regulated by rice differentially expressed genes and rice proteins in the *M. oryzae*-rice protein interaction network. Then the PPI networks of *M. oryzae* and rice were analyzed for GO pathway enrichment, and the modules were separated. Finally, by analyzing the isolated *M. oryzae* and rice protein networks, the main modules’ biological functions and KEGG enrichment pathways were described. The key nodes of *M. oryzae*-rice and their interaction networks were mined by using multi-omics network data to explore the molecular mechanism of *M. oryzae* and rice interaction.

## Results

### Prediction Model Results of the Interspecies Regulatory Protein Pairs Between *M. oryzae* and Rice

**FIGURE 6:**
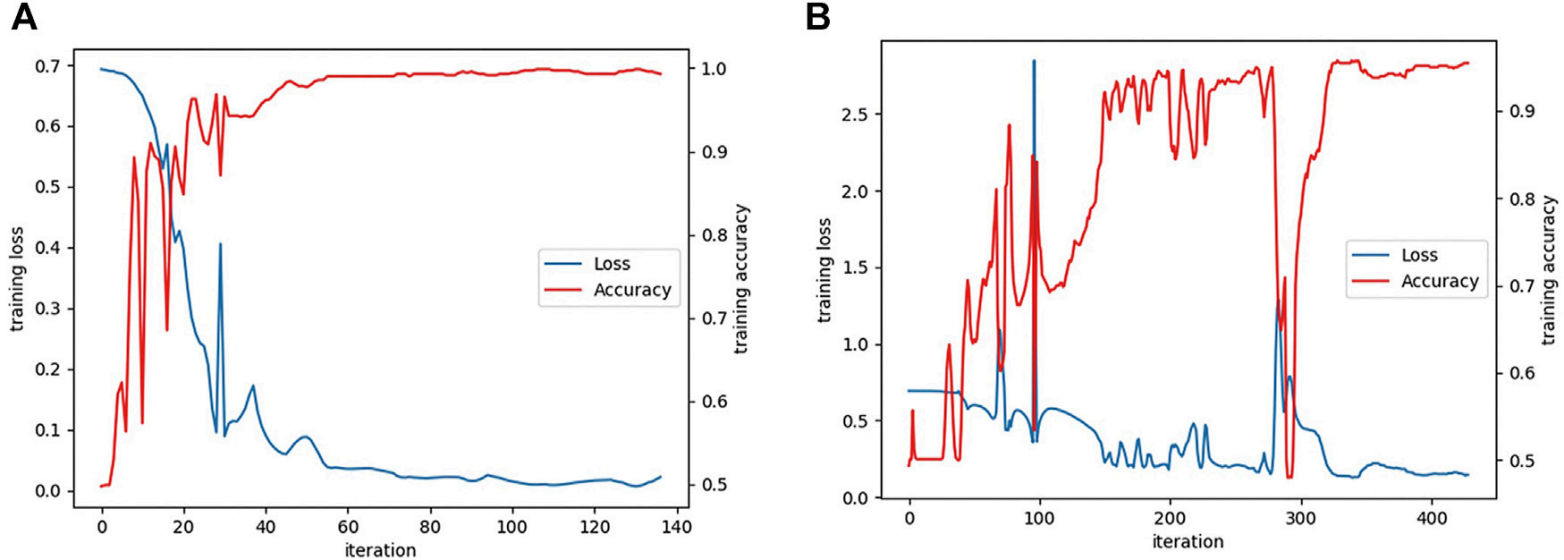
**(A)** The learning curves of textRNN model on functional domain and **(B)** amino acid sequence characteristics.

The learning curves of the textRNN model on the functional domain and amino acid sequence are shown in Figure 6.

The model was evaluated according to the precision, recall and F1 indexes, and the accuracy indexes of the TEXTRNN model in the functional domain and amino acid sequence are shown in Table 1 and Table 2, respectively.

**TABLE 1:** Evaluation indexes of textRNN model on the functional domain.

	Precision	Recall	F1-score	Support
0	0.99	0.99	0.99	420
1	0.99	0.99	0.99	419
Accuracy			0.99	839
Macro avg	0.99	0.99	0.99	839
Weightrd avg	0.99	0.99	0.99	839

**TABLE 2:** Evaluation indexes of textRNN model on the amino acid sequence.

	Precision	Recall	F1-score	Support
0	0.97	0.98	0.98	399
1	0.98	0.97	0.97	398
Accuracy			0.97	797
Macro avg	0.97	0.97	0.97	797
Weightrd avg	0.97	0.97	0.97	797

When textRNN model with the functional domain as feature data was tested, the testAcc of textRNN model was 98.81%, testLoss was 0.029, and the confusion matrix was: $\begin{array}{l} \left[[\text{414}\text{6}\right]\\ \left[\text{4}\text{415}]\right] \end{array}$ .

When textRNN model with protein amino acid sequence as feature data was tested, the testAcc of textRNN model was 97.49%, testLoss was 0.086, and the confusion matrix was: $\begin{array}{l} \left[[\text{391}\text{8}\right]\\ \left[\text{12}\text{386}]\right] \end{array}.$

Therefore, the textRNN model can be used to predict the *M. oryzae*-rice protein interaction pairs, and the prediction model performed well in this paper. Furthermore, the prediction of protein interaction pairs in plants infected by other fungi can also refer to this model.

### Analysis Results of *M. oryzae*-Rice Transcriptome and Proteome Networks

After target prediction of the 623 kinds of *M. oryzae* sRNAs, 1,857 *M. oryzae*-rice sRNA interaction pairs and 664 *M. oryzae* internal sRNA interaction pairs were obtained. By digging positive and negative regulatory factors, 1,166 *M. oryzae* genes, 1,121 rice genes, 1,173 *M. oryzae* proteins and 1,677 rice proteins were found to be involved in the biological process of *M. oryzae* infection to rice. In addition, the transcriptome network of *M. oryzae* and rice was visualized by the Cytoscape tool. There were 20 sRNA-mRNA interaction clusters with two or more sRNAs involved in regulation. The network diagram was shown in Supplementary Figure S1.

Based on the 7,352 *M. oryzae* and rice protein interaction pairs obtained previously, the *M. oryzae*-rice protein interaction network diagram was drawn with a total of 11 rice protein interaction clusters. The network diagram was shown in Supplementary Figure S2.

**FIGURE 7:**
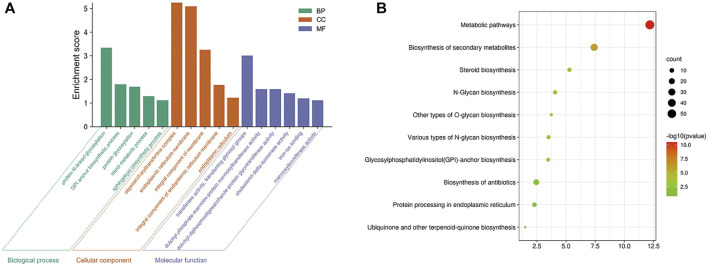
**(A)** Go enrichment module diagram of *M. oryzae*. The first three significantly enriched Go-terms of the biological process modules are protein N-linked glycosylation, GPI anchor biosynthesis process and protein glycosylation. The first three significantly enriched GO-terms of the cell component modules are oligosaccharyltransferase complex, endoplasmic reticulum membrane, and integral component of the membrane. The first three significantly enriched GO-terms of molecular function modules are transferase activity, transferring glycosyl groups, dolichyl-phosphate-mannose-protein mannosyltransferase activity, and dolichyl-diphosphooligosaccharide-protein glycotransferase activity. **(B)** KEGG enrichment bubble diagram of *M. oryzae*. There are 10 significant enrichment pathways in KEGG enrichment pathways, which are mainly related to various glycan organisms, anchor organisms, steroid organisms, biosynthesis of secondary metabolites, protein processing and metabolic pathways.

A total of 593 kinds of *M. oryzae* sRNAs and 581 kinds of *M. oryzae* secreted proteins directly involved in the two interaction mechanisms were excavated through the *M. oryzae*-rice sRNA interaction network and protein interaction network, and they were put into the STRING database for GO pathway enrichment analysis and KEGG enrichment analysis. First, the *p*-value was set as 1E-16, and the GO enrichment results and KEGG enrichment results were derived. The PPI network diagram of *M. oryzae* was too large to be shown in this paper and was shown in Supplementary Figure S3. Next, GO enrichment analysis (Figure 7A) and KEGG pathway enrichment analysis (Figure 7B) were carried out on PPI interaction network diagram of *M. oryzae*. It can be seen that most of these enrichment pathways were involved in the biological processes of sRNA synthesis, protein synthesis and transport in *M. oryzae*. To some extent, the above conclusions proved that *M. oryzae* could complete the infection process of rice through sRNAs and secreted proteins.

**FIGURE 8:**
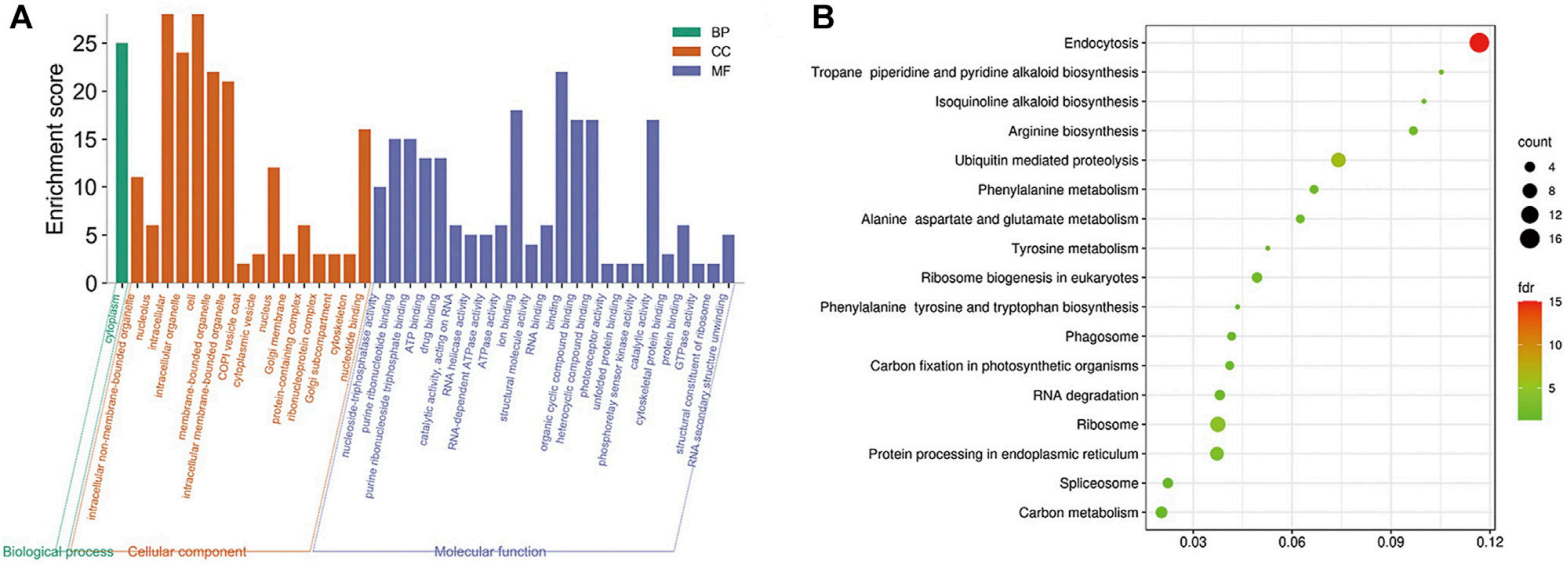
**(A)** Five modules significantly enriched GO functional enrichment module diagram and **(B)** KEGG enrichment pathway bubble diagram of rice.

The obtained rice differentially expressed genes, rice proteins regulated by mRNAs and rice proteins in the protein interaction network of *M. oryzae* and rice were analyzed by GO enrichment and KEGG enrichment. However, there were too many rice-related protein nodes. Firstly, the protein nodes obtained by three ways were mined through the STRING database for their PPI. Then the rice protein interaction pairs obtained were imported into Cytoscape to obtain the rice protein interaction network. Finally, the rice protein interaction network was divided into modules and the largest five rice modules were screened out. The GO enrichment pathways (Figure 8A) and KEGG pathways (Figure 8B) of each module were excavated, respectively.

### Modularity Analysis Results of *M. oryzae* and Rice Regulatory Networks (Cluster 1–10)

In this paper, Clusterviz, a Cytoscape plug-in, was used to segment the protein interaction network between *M. oryzae* and rice into modules, and the FAG-EC algorithm was selected to intercept only the subnet modules with more than six nodes. Next, the segmentation subnet modules were sorted by complexity, and GO function enrichment analysis was carried out for each module. The largest five subnets with significant function enrichment analysis were selected for subsequent analysis and named Cluster 1–10. Then, each subnet’s GO functional modules and KEGG enrichment pathways were mined to explore their biological processes.

Cytoscape calculated the network topology attributes, and its plug-in NetworkAnalyzer was used to calculate the degree and betweenness of nodes in each subnet. Betweenness is a measure of the centrality of a node in the network. In some sense, it measures the influence of a node on information spread through the network. The following formula calculates betweenness:

$$C_{b}\left(n\right)=\underset{s\neq n\neq t}{\sum }\left(\delta_{st}\left(n\right)/\delta_{st}\right)$$

Where s and t are genes different from n in the network, δ_st_ represents the shortest path from s to t, and δ_st_ (n) represents the shortest path from s to t and through n.

The nodes of each subnet were sorted according to betweenness, and the top 6 nodes with the highest betweenness in each subnet were obtained, which were regarded as the central nodes of the subnet and marked in the subnet interaction diagram.

After the segmentation module of the regulatory network of *M. oryzae*, five largest significant functional enrichment subnets were selected, which were the *M. oryzae* helicase activity and protein synthesis module (Cluster 1), *M. oryzae* DNA repair-related module (Cluster 2), *M. oryzae* RNA transport and molecular transport-related module (Cluster 3), *M. oryzae* gene expression and mRNA processing-related module (Cluster 4) and *M. oryzae* biosynthetic pathway-related subnet (Cluster 5). Cluster 1 was closely related to a series of protein synthesis processes and helicase activity (Supplementary Figure S4). The KEGG enrichment pathways of Cluster 2 mainly included nucleotide excision repair pathway, homologous recombination and mismatch repair pathway, etc (Supplementary Figure S5). The KEGG enrichment pathways of Cluster 3 mainly involved RNA transport, MAPK signaling pathway-yeast and endocytosis pathway (Supplementary Figure S6). The GO items of Cluster 4 were mainly involved in RNA transcription, translation and protein synthesis. The KEGG enrichment pathways of Cluster 4 mainly included basic transcription factor enrichment pathway, RNA polymerase enrichment pathway, pyrimidine metabolism enrichment pathway, purine metabolism enrichment pathway, nucleotide excision repair enrichment pathway, ribosome biogenesis in eukaryotes enrichment pathway and metabolic pathway enrichment pathway (Supplementary Figure S7). The KEGG enrichment pathways of Cluster 5 mainly included steroid biosynthesis, antibiotic biosynthesis, secondary metabolite biosynthesis, terpenoid skeleton biosynthesis and metabolic pathway (Supplementary Figure S8).

After the segmentation module of the regulatory network of rice, five largest significant functional enrichment subnets were selected, which were the rice protein binding functional module (Cluster 6), rice GTP and nucleoside triphosphatase-related module (Cluster 7), rice gene expression, transport and metabolism-related module (Cluster 8), rice protein synthesis module (Cluster 9) and rice gene expression and defense response regulation module in rice (Cluster 10). Cluster 6 was significantly enriched in the unfolded protein binding function module (Supplementary Figure S9). Cluster 7 was significantly enriched in GTPase activity, GTP binding and nucleoside-triphosphatase activity (Supplementary Figure S10). The go terms of Cluster 8 were related to regulation of gene expression, transport pathway of biomolecules, and rice metabolic pathways. These GO functional modules showed that the infection process of *M. oryzae* affected the gene expression and metabolism of rice (Supplementary Figure S11). Cluster 9 was significantly enriched in nucleus, ribosome, ribonucleoprotein complex, cytoplasm, cell, translation and structural constituent of ribosome. Most of these GO modules were related to the protein synthesis process (Supplementary Figure 12). The go terms of Cluster 10 were related to regulation of gene expression, protein synthesis, and rice defense module. These GO functional modules showed that the infection process of *M. oryzae* affected the differential gene expression in rice (Supplementary Figure S13).

### PPI Network Analysis and Screening Results of Main Regulatory Factors of *M. oryzae* and Rice

**FIGURE 9:**
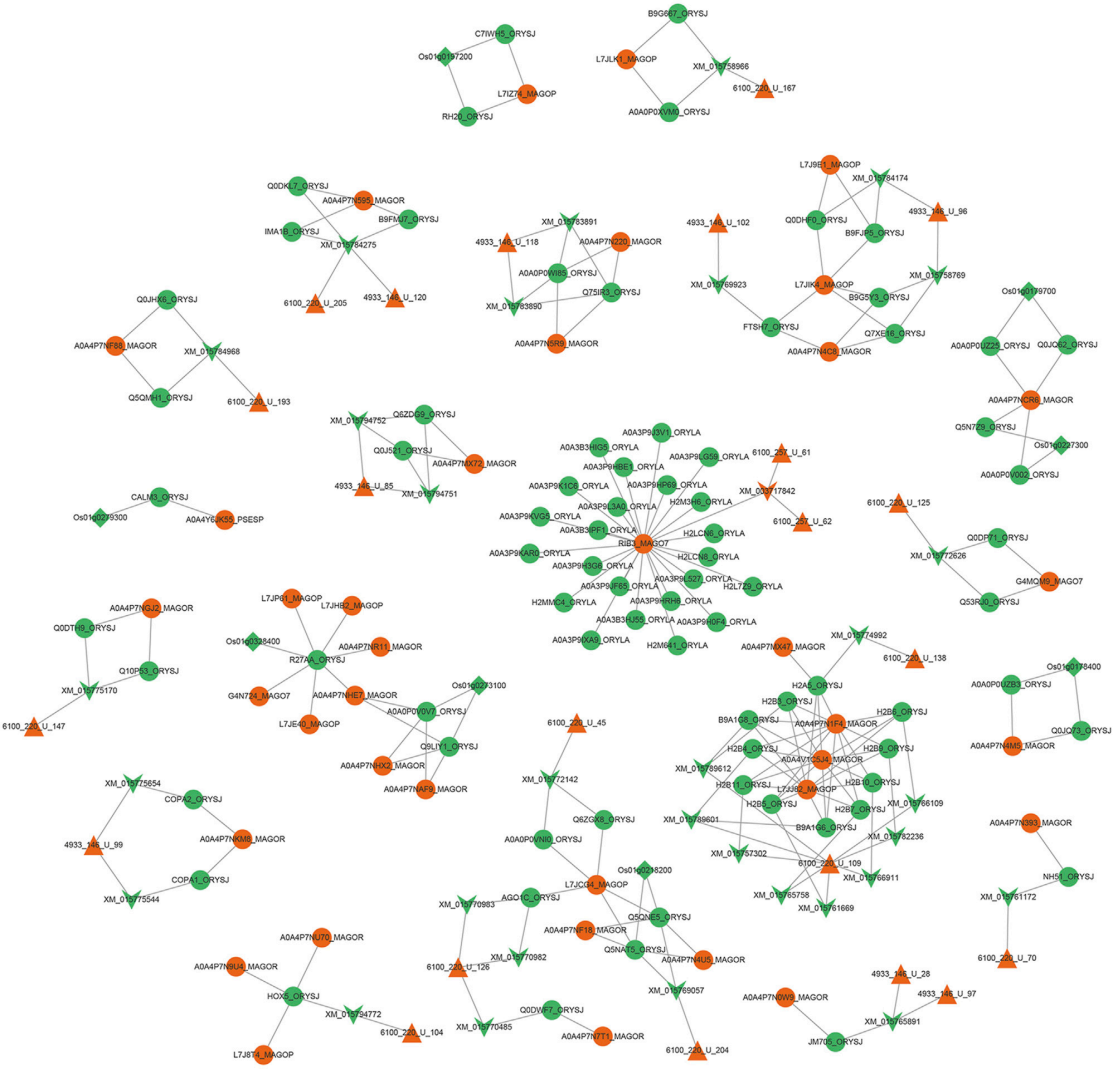
Interaction network diagram between *M. oryzae* and rice main regulatory factors. The red regular triangles are the sRNA nodes of *M. oryzae*, the green inverted triangles are the mRNA nodes of rice, the red inverted triangle is the mRNA node of *M. oryzae*, the green circles are the protein nodes of rice, the red circles are the protein nodes of *M. oryzae*, and the green diamonds are the gene nodes of rice. According to the screening of degree and betweenness, the key protein nodes can be found as RIB3_MAGO7, L7JCG4_MAGOP, A0A4P7NCR6_MAGOR, L7JIK4_MAGOP, HOX5_ORYSJ and R27AA_ORYSJ. The key mRNA nodes can be found as XM_015784275 and XM_015765891. The key sRNA nodes can be found as 4933_146_U_99 and 6100_220_U_126. The key genetic nodes can be found as Os01g0178400 and Os01g0197200.

After the 366 sRNAs up-regulated during the *M. oryzae* infecting rice process to predict the target genes of rice mRNAs, 1,857 rice mRNAs were obtained, pointing to 1,121 rice genes. After the 257 sRNAs were down-regulated during the *M. oryzae* infecting rice process to predict the target genes of *M. oryzae* mRNAs, 664 *M. oryzae* mRNAs were obtained, and 264 *M. oryzae* genes were involved in regulation. The 664 kinds of *M. oryzae* mRNAs were input into the Uniport database to obtain 2,644 protein IDs corresponding to these mRNAs. According to GO, the obtained protein IDs were matched with their interacting protein IDs to expand the proteins involved in regulation by *M. oryzae*. These expanded proteins also used TMHMM to predict secreted proteins, and 337 *M. oryzae* proteins were obtained. Then 601 protein IDs, which were involved in the transboundary regulation of the secreted proteins of *M. oryzae*, were matched with the *M. oryzae*-rice protein interaction pair network to obtain the *M. oryzae* and rice sRNA-protein interaction network (Figure 9).

### Analysis Results of the Core Nodes of the Interaction Network Between *M. oryzae* and Rice

The *M. oryzae* infecting rice interaction network diagram and the rice response of *M. oryzae* infection network diagram obtained above were combined to find the biomolecules that play a role in them. However, the large number of these biomolecules was not conducive to our further analysis of *M. oryzae* and rice interaction mechanism, so core node mining was needed. In this study, the biomolecules involved in the infection of rice by *M. oryzae* were extracted by multi-omics joint analysis, including 8 rice differentially expressed genes, 31 rice mRNAs, 77 rice proteins, 22 *M. oryzae* sRNAs, 1 *M. oryzae* mRNA, and 38 *M. oryzae* proteins (Supplementary Table S1).

22 differentially expressed sRNAs were found, including 12 up-regulated sRNA data of *M. oryzae*, 8 newly increased sRNA data of *M. oryzae*, and 2 down-regulated sRNA data of *M. oryzae*. 20 up-regulated and newly added sRNA data were used to infect rice by targeting rice mRNAs for rice RNA silencing. And 2 down-regulated sRNAs of *M. oryzae* may increase some proteins in *M. oryzae* to achieve the purpose of invading rice by secreted proteins.

**FIGURE 10:**
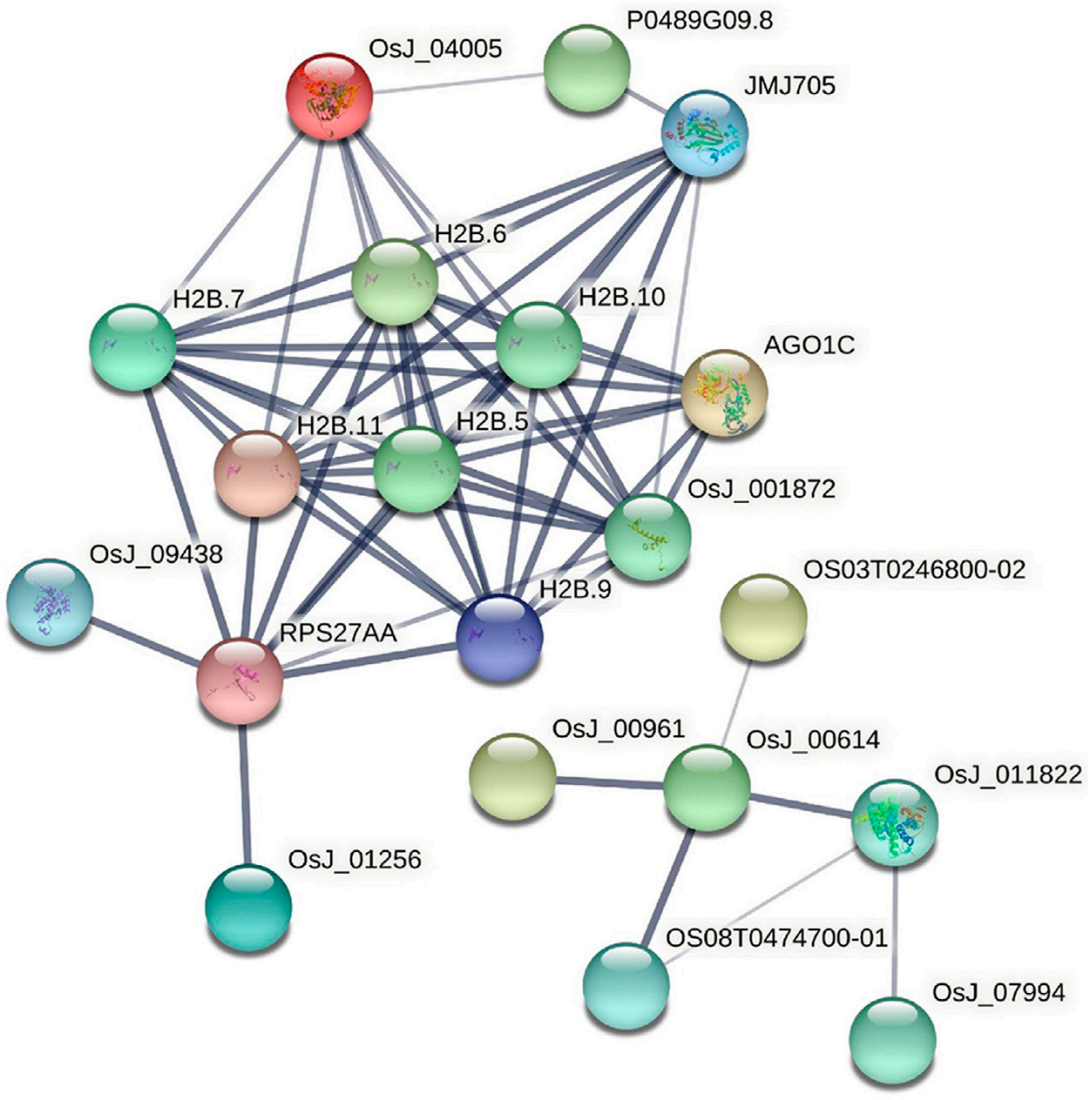
PPI network diagram of rice core nodes.

77 rice core proteins were imported into the STRING database, 32 influential rice gene nodes were obtained, and GO function enrichment analysis and KEGG pathway enrichment analysis was conducted. There were 19 interacting gene nodes. The PPI interaction network diagram of rice core nodes is shown in Figure 10.

The enrichment analysis of the GO pathway of rice core protein nodes found that the significantly enriched GO functions in rice were distributed in three aspects. One was gene expression-related modules, including negative regulation of gene expression, gene expression regulation, epigenetic regulation, gene silencing, and gene expression. The second was protein molecular synthesis and transport-related modules, including protein complex, protein heterodimerization activity, nucleic acid binding, protein binding, organic circular compound binding, heterocyclic compound binding, DNA binding, intracellular protein transport. The third was metabolism-related modules, including protein metabolism process, macromolecular metabolism process, proteolysis, nitrogen compound metabolism process, cellular macromolecular decomposition process, regulation of nitrogen compound metabolism process, regulation of primary metabolic process, primary metabolic process, etc. According to the KEGG pathways enrichment analysis of rice core nodes, the significantly enriched KEGG pathways were protein processing and endocytosis in the endoplasmic reticulum. These GO functional modules with significant enrichment of rice key proteins were basically consistent with the GO functions of the main modules of the rice regulatory network, which verified the accuracy of the rice core proteins mined through multi-omics joint analysis.

**FIGURE 11:**
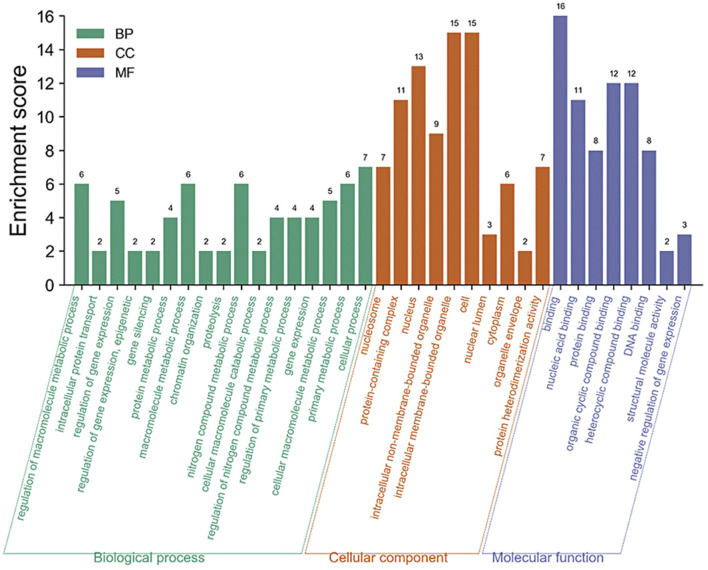
GO pathway enrichment analysis diagram of rice core proteins.

GO functional modules of the *M. oryzae* infecting rice mechanism and rice core nodes for the combined analysis found that the GOs were significantly enriched in the gene expression regulation module, protein synthesis and transport module, and metabolism module. The significant enrichment of gene expression modules indicated that *M. oryzae* silenced rice genes through RNA silencing mechanism to achieve the purpose of infecting rice. In addition, the protein synthesis and transport module showed that *M. oryzae* infected rice by secreted proteins. The module included protein synthesis, nucleic acid binding, protein binding, organic cyclic compound binding, heterocyclic compound binding and transport. These results indicated that *M. oryzae* invaded rice by secreted proteins which combined with some proteins or biomolecules in rice to affect the defense mechanism of rice, thus realizing the infection process. Based on the analysis of KEGG metabolic pathway in rice, it was found that these key proteins in rice affected the metabolic mechanism of rice. After *M. oryzae* infected rice by sRNAs and secreted proteins, the rice metabolism was affected, including nitrogen compound metabolism, protein metabolism, other biological macromolecules metabolism, etc. The table of these GO enrichment modules was shown in Supplementary Table S2. The GO enrichment function diagram of rice core proteins is shown in Figure 11.

## Discussion

In this study, a variety of omics data of *M. oryzae* and rice were used to excavate the interaction network between *M. oryzae* and rice to explore the mechanism of *M. oryzae* infection on rice to mine the key nodes involved in the interaction process. The data of each omics used in this paper included sRNA data before and after *M. oryzae* infecting rice, *M. oryzae* mRNA data, *M. oryzae* protein data, *M. oryzae* gene expression data before and after *M. oryzae* infecting rice, rice mRNA data, rice protein data, and protein data of mode host-mode fungus. First, each omics data was screened separately to mine differentially expressed rice gene data, *M. oryzae*-rice sRNA interaction pairs, and *M. oryzae*-rice protein interaction pairs. Then, the interaction network of each omics was analyzed longitudinally to construct the regulatory network of *M. oryzae*-rice multi-omics interaction and explore its biological process.

In genomics, a total of 1,368 1.5-fold differentially expressed rice genes were extracted by screening the gene expression data of rice before and after the infection of *M. oryzae*. In transcriptomics, this study analyzed the sRNA data of *M. oryzae* before and after infection with rice and obtained 366 kinds of up-regulated and newly added sRNAs of *M. oryzae*, which all had the possibility of interacting with host rice, that is, to infect rice by RNA silencing mechanism. In addition, for the 257 species of *M. oryzae* sRNAs whose expression levels were reduced during the infection process, it may be through the regulation of the protein expression in *M. oryzae*, through the secreted protein into the rice to achieve the purpose of infection. Therefore, according to the two infection mechanisms of *M. oryzae*, the 623 kinds of *M. oryzae* sRNAs screened were analyzed. Furthermore, through the method of target gene prediction, 1,857 sRNA interaction pairs of *M. oryzae*-rice and 664 sRNA interaction pairs of *M. oryzae* were found.

In proteomics, some studies have proved that the secreted proteins of the pathogen can enter the host body and interact with the host proteins to interfere with the protein expression of the host. However, it is not clear which protein molecules are involved in the infection process of *M. oryzae* to affect the defense and growth of rice in the existing studies. In this paper, the protein interaction pairs between mode pathogens and mode hosts that experiments have verified were collected and used as the prediction template. Firstly, the interolog method based on homology was used to predict the protein interaction pairs between *M. oryzae* and rice. Next, the domain-domain method was used to make the second prediction of the protein interaction pairs predicted by the interolog method. Then TMHMM secreted protein prediction tool was used to screen the secreted proteins of *M. oryzae*. In the screening of the final protein interaction pairs, the three prediction methods should be met simultaneously, and 7,352 protein interaction pairs of *M. oryzae*-rice were obtained.

In this study, a total of 8 rice differentially expressed genes, 31 rice mRNAs, 77 rice proteins, 22 *M. oryzae* sRNAs, 1 *M. oryzae* mRNA and 38 *M. oryzae* proteins were identified as the core nodes of the *M. oryzae* and rice multi-omics interaction network by high-throughput data analysis, combined with joint analysis of *M. oryzae* and rice multi-omics data, which involved significantly enriched GO modules. Most of them were related to gene expression, molecular protein synthesis, molecular transport and metabolism, that is, the infection mechanism of *M. oryzae*. However, all the experiments in this paper were based on the premise that sRNA and protein interaction mechanisms exist between *M. oryzae* and rice. The accuracy of this experiment still needs to be further verified. In addition, due to the mutual regulation between plants and pathogens, some host sRNAs and secreted proteins can enter the fungi during the infection process to resist infection. However, this paper only studied the infection mechanism of *M. oryzae* and neglected the analysis of the defense mechanism of rice. Moreover, significant enrichment of biomolecular transport modules was found in the GO function enrichment analysis of key factors of *M. oryzae* in this study, but it is not clear which rice biomolecules are involved in the defense mechanism. And although there are some insufficient, this paper for the *M. oryzae* infecting rice joint analysis of multi-omics data, which provided a specific data basis for further study of the mechanism of *M. oryzae*-rice interaction, made some specific contributions to the prevention of diseases and insect pests in rice and provided a new train of thought and theoretical basis for the fungus-plant interactions mechanism research.

## Data Availability

Publicly available datasets were analyzed in this study. This data can be found here: https://www.ncbi.nlm.nih.gov/geo/query/acc.cgi?acc=GSE110088; https://www.ncbi.nlm.nih.gov/geo/query/acc.cgi?acc=GPL2025; https://rapdb.dna.affrc.go.jp/download/irgsp1.html; https://www.ncbi.nlm.nih.gov/Traces/study/?acc=SRX214117; https://www.ncbi.nlm.nih.gov/Traces/study/?acc=SRX214123; https://www.ncbi.nlm.nih.gov/geo/query/acc.cgi?acc=GSM973470; https://www.ncbi.nlm.nih.gov/geo/query/acc.cgi?acc=GSM973471; https://www.ncbi.nlm.nih.gov/Traces/wgs/AACU03?val=AACU03.1; https://www.ncbi.nlm.nih.gov/Traces/wgs/AACU03?val=LVCG01.1; https://hpidb.igbb.msstate.edu/about.html#stats.
