# Supplementary material for: Construction of sRNA Regulatory Network for Magnaporthe oryzae Infecting Rice Based on Multi-Omics Data
Source: Front Genet. 2021 Nov 12;12:763915. doi: 10.3389/fgene.2021.763915 (PMC8633311; doi:10.3389/fgene.2021.763915)
Supplement: Supplementary file 1 [file Image12.PDF]

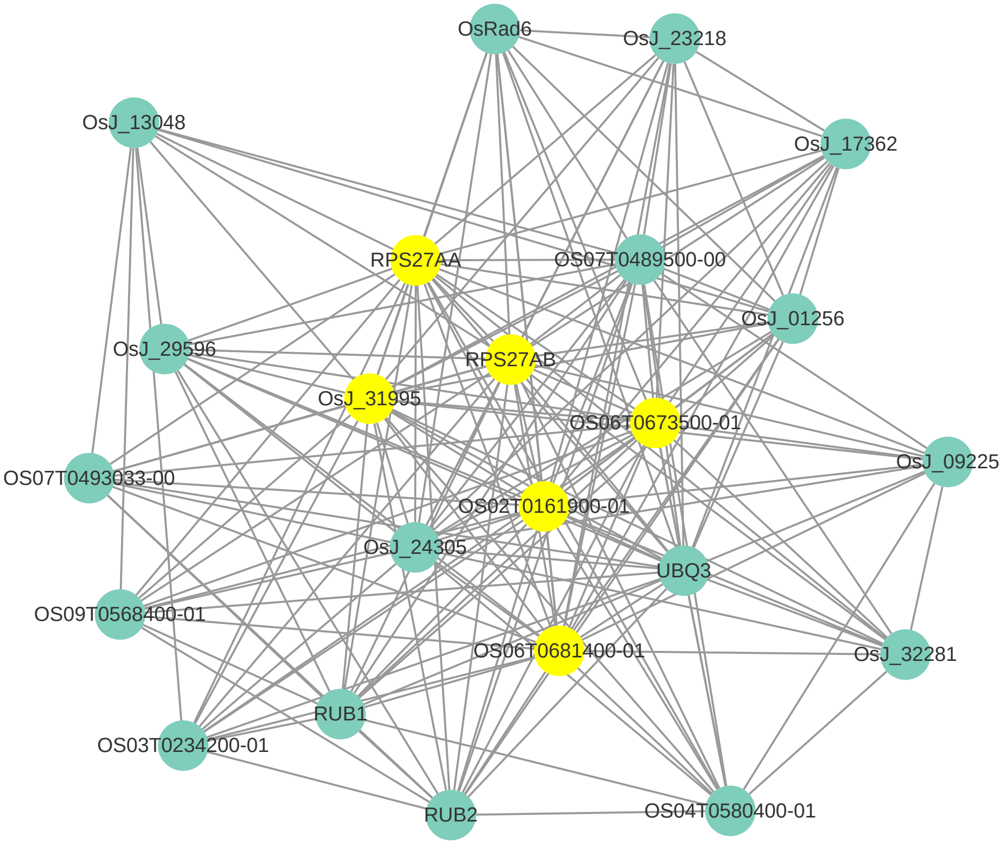

Supplementary Figure 12. Rice protein synthesis module (Cluster 9). Cluster 9 contains 23 gene nodes. In this section, the betweenness of each node is calculated according to the network topology attribute calculation method and sorted according to its criticality to nodes. The top 6 genes in betweeness ranking are selected as the central regulatory genes in Cluster 9, which are RPS27AA, RPS27AB, OsJ_31995, OS02T0161900-01, OS06T0673500-01, OS06T0681400-01, the genes with central regulatory function shown as yellow nodes in the network diagram.

The main modules of this network are apparently enriched in the following modules, including nucleus, ribosome, ribonucleoprotein complex, cytoplasm, cell, translation and structural constituent of ribosome. Most of these GO modules are related to the protein synthesis process.
