# Supplementary material for: Construction of sRNA Regulatory Network for Magnaporthe oryzae Infecting Rice Based on Multi-Omics Data
Source: Front Genet. 2021 Nov 12;12:763915. doi: 10.3389/fgene.2021.763915 (PMC8633311; doi:10.3389/fgene.2021.763915)
Supplement: Supplementary file 3 [file Table1.DOCX]

**Supplementary Table 1.** The core nodes of M. oryzae.

| **ID** | **Type** |
| --- | --- |
| Os01g0178400 | Rice differentially expressed gene |
| Os01g0179700 | Rice differentially expressed gene |
| Os01g0197200 | Rice differentially expressed gene |
| Os01g0218200 | Rice differentially expressed gene |
| Os01g0227300 | Rice differentially expressed gene |
| Os01g0273100 | Rice differentially expressed gene |
| Os01g0279300 | Rice differentially expressed gene |
| Os01g0328400 | Rice differentially expressed gene |
| XM_015794752 | Rice mRNA |
| XM_015794751 | Rice mRNA |
| XM_015770485 | Rice mRNA |
| XM_015775654 | Rice mRNA |
| XM_015775544 | Rice mRNA |
| XM_015782236 | Rice mRNA |
| XM_015757302 | Rice mRNA |
| XM_015766109 | Rice mRNA |
| XM_015765758 | Rice mRNA |
| XM_015789612 | Rice mRNA |
| XM_015789601 | Rice mRNA |
| XM_015774992 | Rice mRNA |
| XM_015772142 | Rice mRNA |
| XM_015783891 | Rice mRNA |
| XM_015783890 | Rice mRNA |
| XM_015772626 | Rice mRNA |
| XM_015775170 | Rice mRNA |
| XM_015784174 | Rice mRNA |
| XM_015770983 | Rice mRNA |
| XM_015770982 | Rice mRNA |
| XM_015758966 | Rice mRNA |
| XM_015794772 | Rice mRNA |
| XM_015761669 | Rice mRNA |
| XM_015766911 | Rice mRNA |
| XM_015784275 | Rice mRNA |
| XM_015761172 | Rice mRNA |
| XM_015758769 | Rice mRNA |
| XM_015769923 | Rice mRNA |
| XM_015784968 | Rice mRNA |
| XM_015769057 | Rice mRNA |
| XM_015765891 | Rice mRNA |
| A0A0P0UZB3_ORYSJ | Rice protein |
| Q0JQ73_ORYSJ | Rice protein |
| A0A0P0UZ25_ORYSJ | Rice protein |
| Q0JQ62_ORYSJ | Rice protein |
| RH20_ORYSJ | Rice protein |
| C7IWH5_ORYSJ | Rice protein |
| Q5NAT5_ORYSJ | Rice protein |
| Q5QNE5_ORYSJ | Rice protein |
| A0A0P0V002_ORYSJ | Rice protein |
| Q5N7Z9_ORYSJ | Rice protein |
| A0A0P0V0V7_ORYSJ | Rice protein |
| Q9LIY1_ORYSJ | Rice protein |
| CALM3_ORYSJ | Rice protein |
| R27AA_ORYSJ | Rice protein |
| Q0J521_ORYSJ | Rice protein |
| Q6ZDG9_ORYSJ | Rice protein |
| Q0DWF7_ORYSJ | Rice protein |
| COPA2_ORYSJ | Rice protein |
| COPA1_ORYSJ | Rice protein |
| H2B9_ORYSJ | Rice protein |
| H2B11_ORYSJ | Rice protein |
| H2B6_ORYSJ | Rice protein |
| H2B7_ORYSJ | Rice protein |
| H2B3_ORYSJ | Rice protein |
| B9A1G8_ORYSJ | Rice protein |
| H2B4_ORYSJ | Rice protein |
| B9A1G6_ORYSJ | Rice protein |
| H2A5_ORYSJ | Rice protein |
| A0A0P0VNI0_ORYSJ | Rice protein |
| Q6ZGX8_ORYSJ | Rice protein |
| A0A0P0WI85_ORYSJ | Rice protein |
| Q75IR3_ORYSJ | Rice protein |
| Q0DP71_ORYSJ | Rice protein |
| Q53RJ0_ORYSJ | Rice protein |
| Q0DTH9_ORYSJ | Rice protein |
| Q10P53_ORYSJ | Rice protein |
| B9FJP5_ORYSJ | Rice protein |
| Q0DHF0_ORYSJ | Rice protein |
| AGO1C_ORYSJ | Rice protein |
| A0A0P0XVM0_ORYSJ | Rice protein |
| B9G667_ORYSJ | Rice protein |
| HOX5_ORYSJ | Rice protein |
| H2B5_ORYSJ | Rice protein |
| H2B10_ORYSJ | Rice protein |
| IMA1B_ORYSJ | Rice protein |
| B9FMJ7_ORYSJ | Rice protein |
| Q0DKL7_ORYSJ | Rice protein |
| NH51_ORYSJ | Rice protein |
| B9G5Y3_ORYSJ | Rice protein |
| Q7XE16_ORYSJ | Rice protein |
| FTSH7_ORYSJ | Rice protein |
| Q0JHX6_ORYSJ | Rice protein |
| Q5QMH1_ORYSJ | Rice protein |
| JM705_ORYSJ | Rice protein |
| A0A3P9JF65_ORYLA | Rice protein |
| A0A3P9L3A0_ORYLA | Rice protein |
| H2MMC4_ORYLA | Rice protein |
| A0A3P9KAR0_ORYLA | Rice protein |
| H2LCN6_ORYLA | Rice protein |
| A0A3P9IXA9_ORYLA | Rice protein |
| A0A3B3HIG5_ORYLA | Rice protein |
| H2L7Z9_ORYLA | Rice protein |
| A0A3P9HRH6_ORYLA | Rice protein |
| H2LCN8_ORYLA | Rice protein |
| A0A3P9LG59_ORYLA | Rice protein |
| A0A3B3IPF1_ORYLA | Rice protein |
| H2M641_ORYLA | Rice protein |
| H2M3H6_ORYLA | Rice protein |
| A0A3P9H0F4_ORYLA | Rice protein |
| A0A3P9J3V1_ORYLA | Rice protein |
| A0A3P9HBE1_ORYLA | Rice protein |
| A0A3P9KVG5_ORYLA | Rice protein |
| A0A3P9L527_ORYLA | Rice protein |
| A0A3P9H3G6_ORYLA | Rice protein |
| A0A3P9HP69_ORYLA | Rice protein |
| A0A3B3HJ55_ORYLA | Rice protein |
| A0A3P9K1C6_ORYLA | Rice protein |
| XM_003717842 | M. oryzae mRNA |
| A0A4P7N4M5_MAGOR | M. oryzae protein |
| A0A4P7NCR6_MAGOR | M. oryzae protein |
| L7IZ74_MAGOP | M. oryzae protein |
| A0A4P7NF18_MAGOR | M. oryzae protein |
| A0A4P7NHE7_MAGOR | M. oryzae protein |
| A0A4P7NHX2_MAGOR | M. oryzae protein |
| A0A4P7NAF9_MAGOR | M. oryzae protein |
| A0A4Y6JK55_PSESP | M. oryzae protein |
| G4N724_MAGO7 | M. oryzae protein |
| L7JE40_MAGOP | M. oryzae protein |
| L7JHB2_MAGOP | M. oryzae protein |
| L7JP61_MAGOP | M. oryzae protein |
| A0A4P7NR11_MAGOR | M. oryzae protein |
| A0A4P7MX72_MAGOR | M. oryzae protein |
| A0A4P7N7T1_MAGOR | M. oryzae protein |
| A0A4P7NKM8_MAGOR | M. oryzae protein |
| A0A4V1C5J4_MAGOR | M. oryzae protein |
| A0A4P7N1F4_MAGOR | M. oryzae protein |
| L7JJ82_MAGOP | M. oryzae protein |
| L7JCG4_MAGOP | M. oryzae protein |
| A0A4P7MX47_MAGOR | M. oryzae protein |
| A0A4P7N220_MAGOR | M. oryzae protein |
| G4MQM9_MAGO7 | M. oryzae protein |
| A0A4P7NGJ2_MAGOR | M. oryzae protein |
| L7JIK4_MAGOP | M. oryzae protein |
| L7J9E1_MAGOP | M. oryzae protein |
| L7JLK1_MAGOP | M. oryzae protein |
| A0A4P7N9U4_MAGOR | M. oryzae protein |
| A0A4P7N595_MAGOR | M. oryzae protein |
| A0A4P7N393_MAGOR | M. oryzae protein |
| A0A4P7NU70_MAGOR | M. oryzae protein |
| A0A4P7N4C8_MAGOR | M. oryzae protein |
| L7J8T4_MAGOP | M. oryzae protein |
| A0A4P7NF88_MAGOR | M. oryzae protein |
| A0A4P7N4U5_MAGOR | M. oryzae protein |
| A0A4P7N0W9_MAGOR | M. oryzae protein |
| A0A4P7N5R9_MAGOR | M. oryzae protein |
| RIB3_MAGO7 | M. oryzae protein |
| **M. oryzae sRNA_ID** | **Seq** |
| 6100_257_U_62 | TGCGGGCGGGCGGTCTGGATA |
| 6100_257_U_61 | TACGACCTGAGGAGGACGCTGGGCG |
| 6100_220_U_70 | GGGTGGAGCCTGCGGCTT |
| 6100_220_U_45 | GGAATCGGATTCGGAACC |
| 6100_220_U_205 | GAGCGCTGCAGTGCCTGAA |
| 6100_220_U_204 | AGTGGTCGTAGACCGCCTGA |
| 6100_220_U_193 | TAGGATTTGGATCATCTCGG |
| 6100_220_U_167 | GACTAGGGATCGGACGGT |
| 6100_220_U_147 | GAGTATTTGGACGATTTCT |
| 6100_220_U_138 | TAGAGGCGTGGACGGACAA |
| 6100_220_U_126 | ATGGCAGAACGTCCGAACA |
| 6100_220_U_125 | CAGGCGAGGGCGCTCTGCT |
| 6100_220_U_109 | GGACATGGTTTTGGACGAA |
| 6100_220_U_104 | AGGGCGAGCTCCTGTTTCTGCT |
| 4933_146_U_99 | GACAAGAATGGTGGACGAAA |
| 4933_146_U_97 | GGACCGGACCATGGCAGAT |
| 4933_146_U_96 | TAGCGGGGAACTGTGCATG |
| 4933_146_U_85 | TACAACGTCGAGGCTCAGAAGGCT |
| 4933_146_U_28 | GCTCTGGGTGAGAACATTGGCAT |
| 4933_146_U_120 | AGCGCCAACACAGAGCCTGA |
| 4933_146_U_118 | TGGAACATTACGAACCAGA |
| 4933_146_U_102 | ACCGTCTGCTGCACGATAGTG |
