# Supplementary material for: Construction of sRNA Regulatory Network for Magnaporthe oryzae Infecting Rice Based on Multi-Omics Data
Source: Front Genet. 2021 Nov 12;12:763915. doi: 10.3389/fgene.2021.763915 (PMC8633311; doi:10.3389/fgene.2021.763915)
Supplement: Supplementary file 4 [file Image9.PDF]

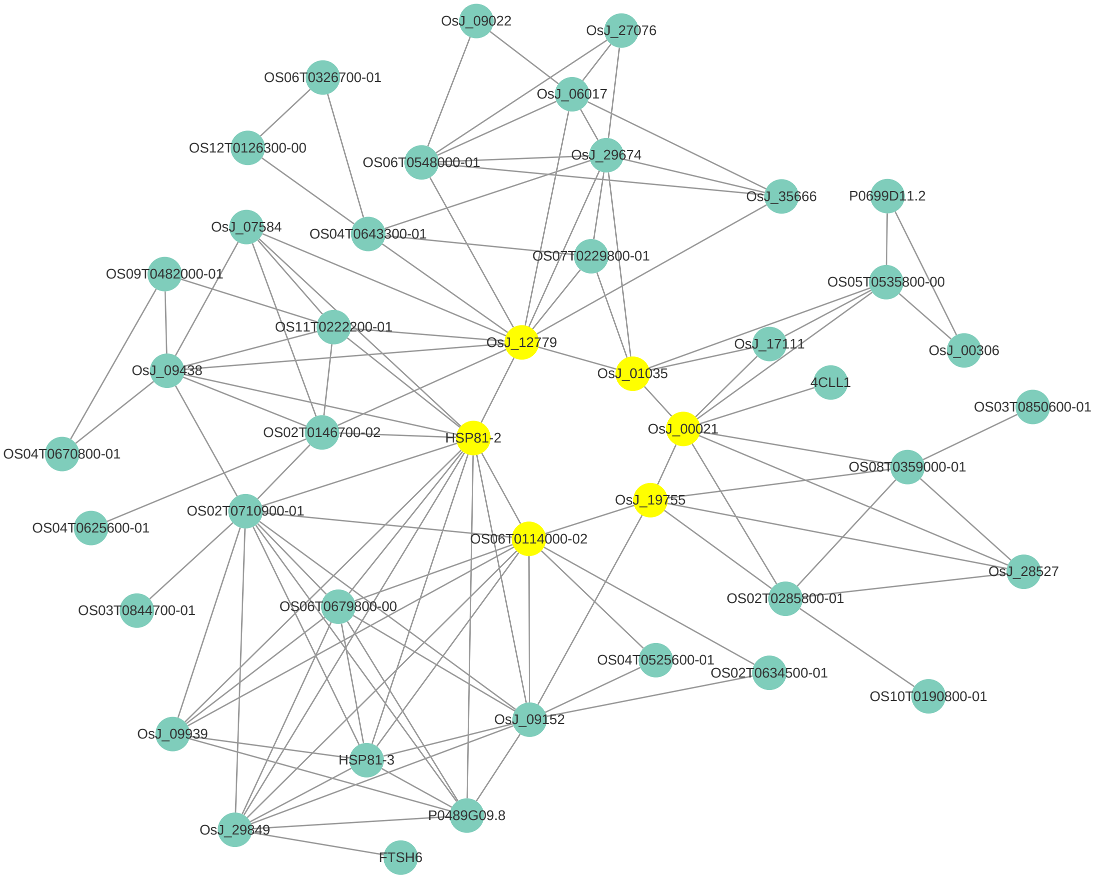

Supplementary Figure 9. Rice protein binding functional module (Cluster 6). Cluster 6 contains 44 gene nodes. In this section, the betweenness of each node is calculated according to the network topology attribute calculation method and sorted according to its criticality to nodes. The top 6 genes in betweeness ranking are selected as the central regulatory genes in Cluster 6, which are OsJ_12779, HSP81-2, OsJ_01035, OsJ_00021, OsJ_19755, OS06T0114000-02, the genes with central regulatory function shown as yellow nodes in the network diagram.
The network module is mainly enriched in the unfold protein binding (GO:0051082) function module, with Hsp81-2 and Hsp81-3 involved in regulation, of which Hsp81-2 is also the central regulator of the network module.
