# Supplementary material for: Construction of sRNA Regulatory Network for Magnaporthe oryzae Infecting Rice Based on Multi-Omics Data
Source: Front Genet. 2021 Nov 12;12:763915. doi: 10.3389/fgene.2021.763915 (PMC8633311; doi:10.3389/fgene.2021.763915)
Supplement: Supplementary file 5 [file Image10.PDF]

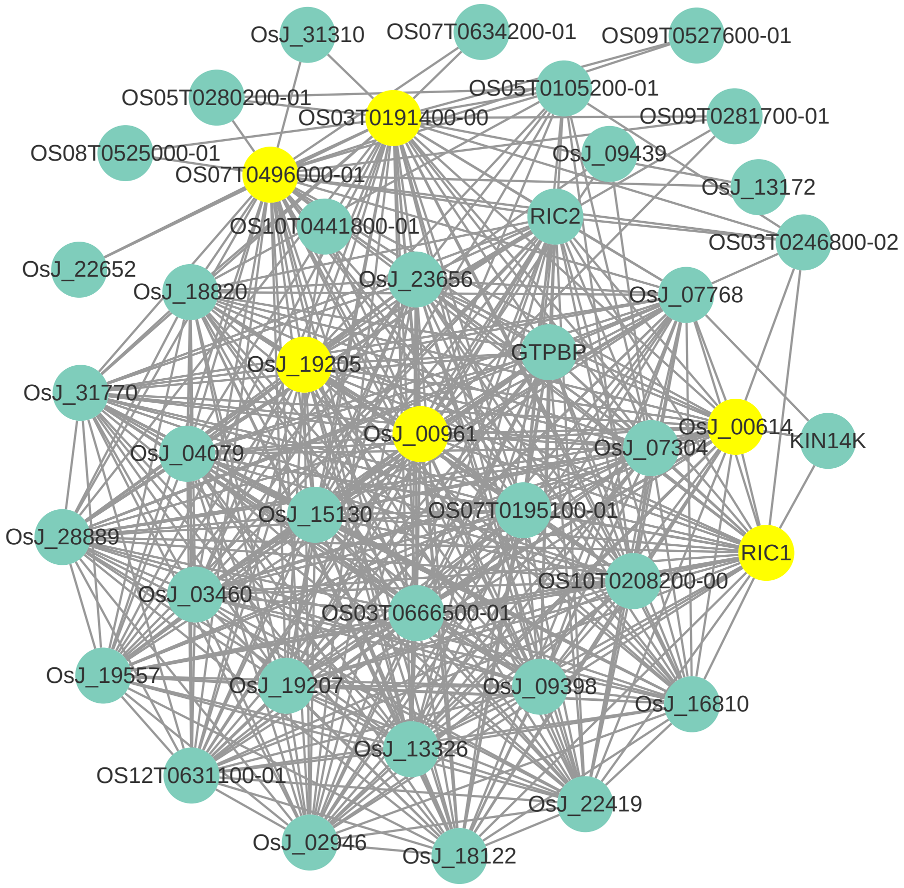

Supplementary Figure 10. Rice GTP and nucleoside-triphosphatase related module (Cluster 7). Cluster 7 contains 42 gene nodes. In this section, the betweenness of each node is calculated according to the network topology attribute calculation method and sorted according to its criticality to nodes. The top 6 genes in betweeness ranking are selected as the central regulatory genes in Cluster 7, which are OS03T0191400-00, OS07T0496000-01, OsJ_19205, OsJ_00961, OsJ_00614, RIC1, the genes with central regulatory function shown as yellow nodes in the network diagram.

This network module mainly focuses on GTPase activity (GO:0003924), GTP binding (GO: 0005525) and nucleoside-triphosphatase activity (GO:0017111). The genes involved in regulation are RIC1,KIN14K and RIC2, among which, RIC1 is also the central regulator of the network module. Nucleoside-triphosphatase activity is an important link that restricts the translation of mRNA into protein. GTP binding proteins are involved in a variety of life activities of cells, such as cell communication, ribosomal and endoplasmic reticulum binding, vesicle transport, protein synthesis, etc.
