# Supplementary material for: Construction of sRNA Regulatory Network for Magnaporthe oryzae Infecting Rice Based on Multi-Omics Data
Source: Front Genet. 2021 Nov 12;12:763915. doi: 10.3389/fgene.2021.763915 (PMC8633311; doi:10.3389/fgene.2021.763915)
Supplement: Supplementary file 6 [file Image6.PDF]

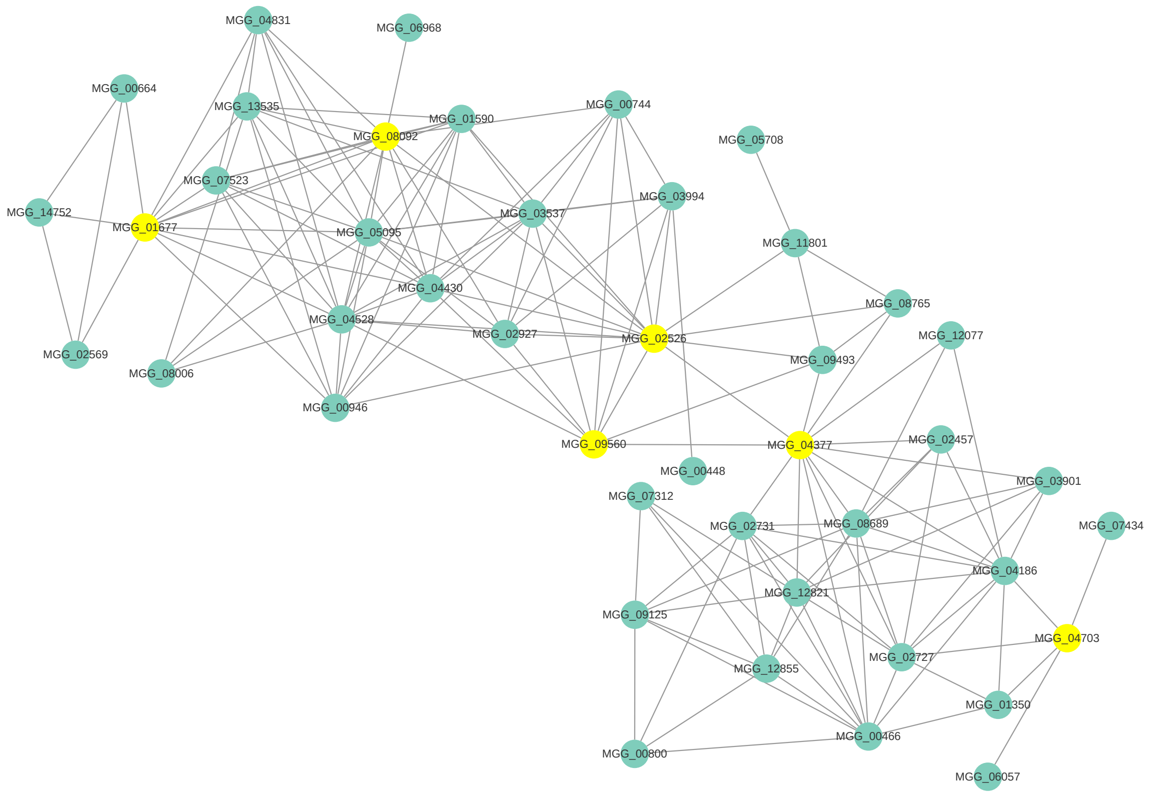

Supplementary Figure 6. M. oryzae RNA transport and molecular transport-related module (Cluster 3). Cluster 3 contains 44 gene nodes. In this section, the betweenness of each node is calculated according to the network topology attribute calculation method and sorted according to its criticality to nodes. The top 6 genes in betweeness ranking are selected as the central regulatory genes in Cluster 3, which are MGG_ 04377, MGG_02526, MGG_01677, MGG_09560, MGG_04703, MGG_08092, the genes with central regulatory function shown as yellow nodes in the network diagram. There is no apparent enrichment of GO module, so this section only analyzes Cluster 3 through KEGG enrichment analysis. There are apparently enriched 6 KEGG enrichment pathways in Cluster 3. There are 19 genes involved in RNA transport, MAPK signaling pathway-yeast and endocytosis pathway in the KEGG enrichment pathway, which account for 43% of the nodes in Cluster 3.
