# Supplementary material for: Construction of sRNA Regulatory Network for Magnaporthe oryzae Infecting Rice Based on Multi-Omics Data
Source: Front Genet. 2021 Nov 12;12:763915. doi: 10.3389/fgene.2021.763915 (PMC8633311; doi:10.3389/fgene.2021.763915)
Supplement: Supplementary file 7 [file Image13.PDF]

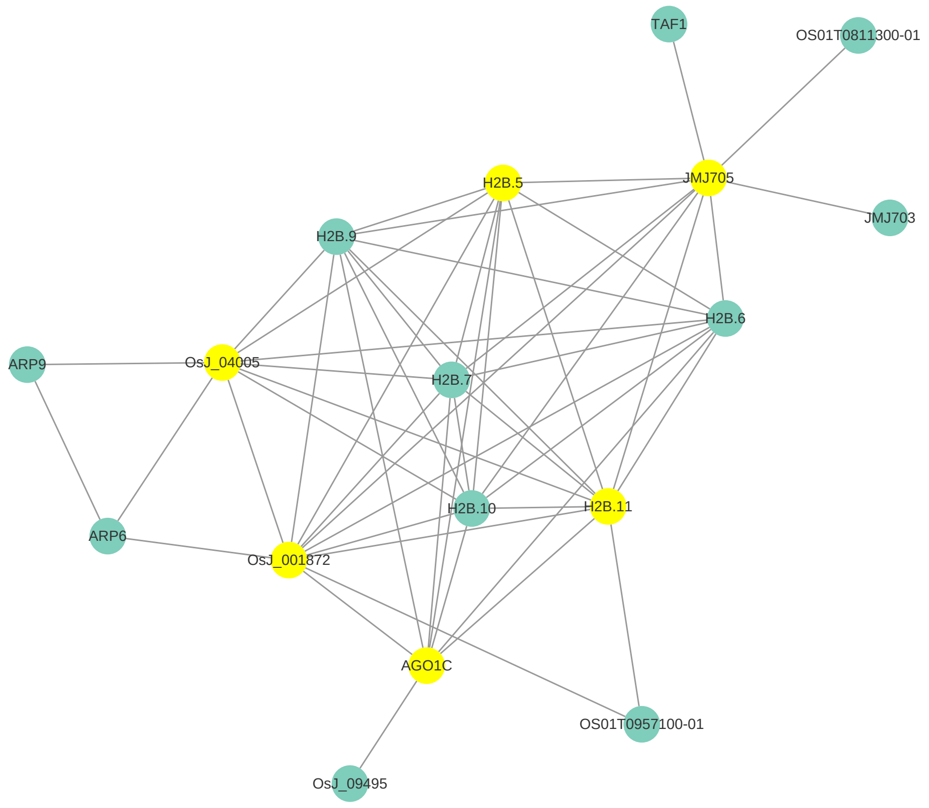

Supplementary Figure 13. Rice gene expression and defense response regulation module (Cluster 10). Cluster 10 contains 23 gene nodes. In this section, the betweenness of each node is calculated according to the network topology attribute calculation method and sorted according to its criticality to nodes. The top 6 genes in betweeness ranking are selected as the central regulatory genes in Cluster 10, which are H2B.5, JMJ705, OsJ_04005, H2B.11, OsJ_001872, AGO1C, the genes with central regulatory function shown as yellow nodes in the network diagram.

From the results of the apparent enrichment of GO in this network module, it can be seen that one of the functions of the regulatory module is related to gene expression regulation, such as positive regulation of gene expression, negative regulation of gene expression and epigenetic, gene silencing, etc. The second is related to protein synthesis, such as protein-containing complex, protein heterodimerization activity, protein binding, histone demethylase activity, etc.. The third is related to the defense module of rice, such as defense response. These GO functional modules showed that the infection process of M. oryzae affected the differential gene expression in rice. The GO module (GO:0006952) involved in the defense process of rice is excavated, in which ARP6 and JMJ705 are involved in the defense process of rice. JMJ705 is also the central regulator of the network module.
