# Supplementary material for: Construction of sRNA Regulatory Network for Magnaporthe oryzae Infecting Rice Based on Multi-Omics Data
Source: Front Genet. 2021 Nov 12;12:763915. doi: 10.3389/fgene.2021.763915 (PMC8633311; doi:10.3389/fgene.2021.763915)
Supplement: Supplementary file 8 [file Image8.PDF]

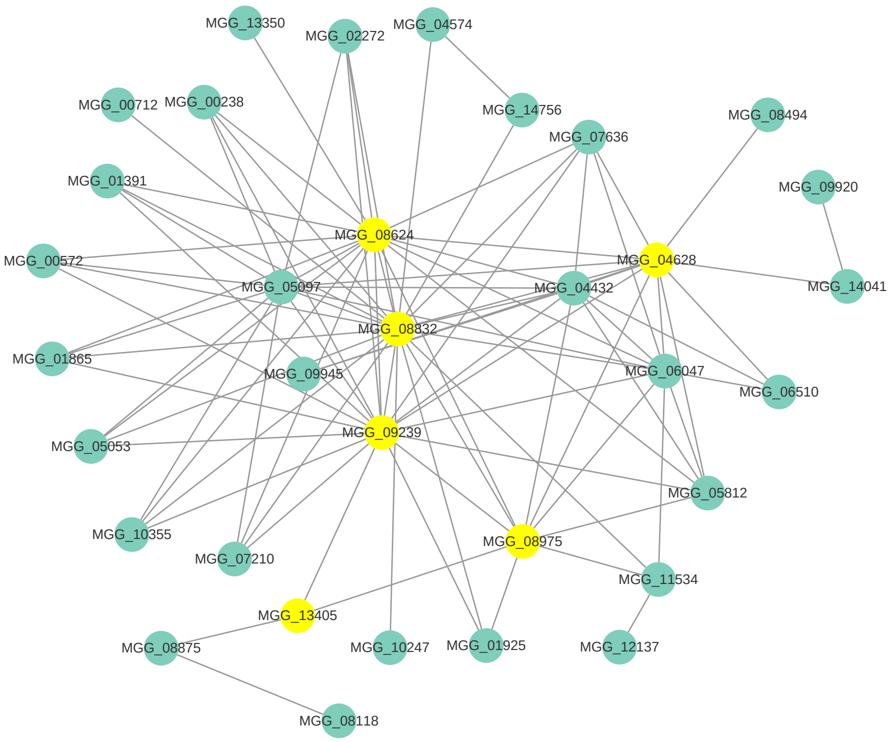

Supplementary Figure 8. M. oryzae biosynthetic pathway-related subnet (Cluster 5). Cluster 5 contains 34 gene nodes. In this section, the betweenness of each node is calculated according to the network topology attribute calculation method and sorted according to its criticality to nodes. The top 6 genes in betweeness ranking are selected as the central regulatory genes in Cluster 5, which are MGG_08832, MGG_04628, MGG_09239, MGG_08624, MGG_13405, MGG_08975, the genes with central regulatory function shown as yellow nodes in the network diagram.

There are 6 apparently enriched KEGG enrichment pathways in this subnet, and the KEGG enrichment pathways are arranged from small to large according to the p-value as follows: steroid biosynthesis, antibiotic biosynthesis, secondary metabolite biosynthesis, terpenoid skeleton biosynthesis and metabolic pathway.
