# Supplementary material for: Construction of sRNA Regulatory Network for Magnaporthe oryzae Infecting Rice Based on Multi-Omics Data
Source: Front Genet. 2021 Nov 12;12:763915. doi: 10.3389/fgene.2021.763915 (PMC8633311; doi:10.3389/fgene.2021.763915)
Supplement: Supplementary file 9 [file Image4.PDF]

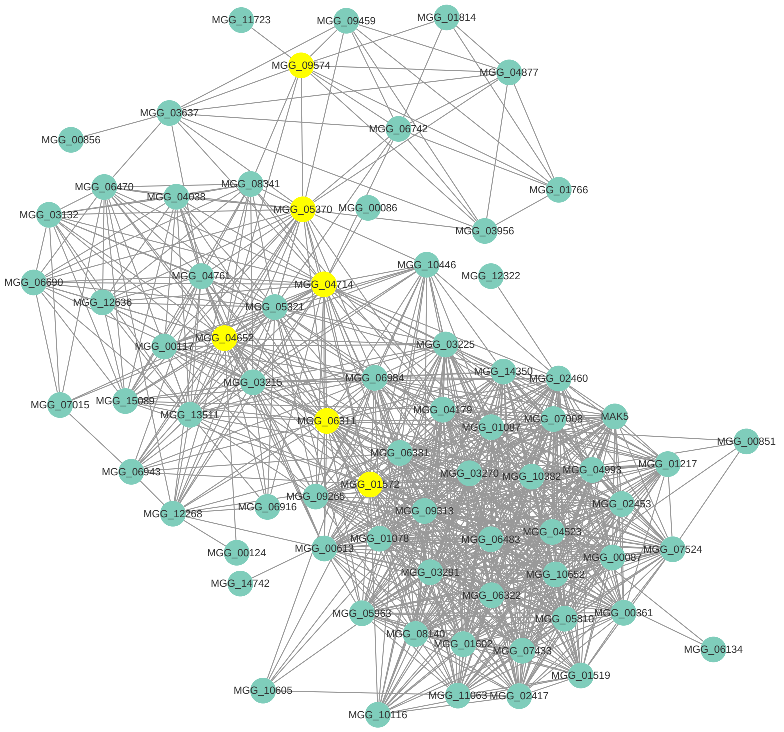

Supplementary Figure 4. M. oryzae helicase activity and protein synthesis module (Cluster 1). Cluster 1 contains 74 gene nodes. In this section, the betweenness of each node is calculated according to the network topology attribute calculation method and sorted according to its criticality to nodes. The top 6 genes in betweeness ranking are selected as the central regulatory genes in Cluster 1, which are MGG_04714, MGG_05370, MGG_01572, MGG_04652, MGG_ 09574, MGG_06311, the genes with central regulatory function shown as yellow nodes in the network diagram.

This network module is closely associated with a range of protein synthesis processes, including rRNA processing (GO:0006364), ribosomal large subunit biogenesis (GO:0042273), RNA binding (RNA binding, GO:0019843), ATP binding (GO:0005524), and Cluster 1 is also closely related to helicase activity (GO:0003824). The main genes involved in regulation are MGG_09313, MGG_10652, MGG_02460, MGG_04179, MGG_05810, MGG_08140, MAK5, MGG_04523, MGG_04993, MGG_06483.
