# Supplementary material for: Construction of sRNA Regulatory Network for Magnaporthe oryzae Infecting Rice Based on Multi-Omics Data
Source: Front Genet. 2021 Nov 12;12:763915. doi: 10.3389/fgene.2021.763915 (PMC8633311; doi:10.3389/fgene.2021.763915)
Supplement: Supplementary file 10 [file Image2.PDF]

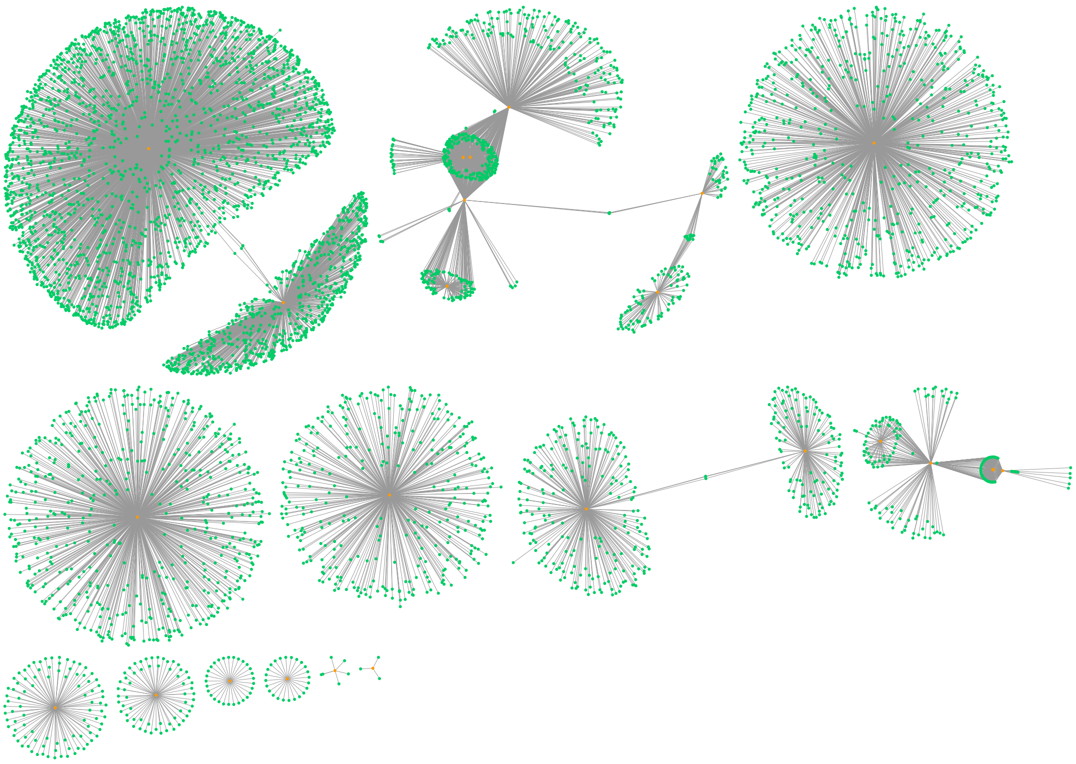

Supplementary Figure 2. M. oryzae-rice protein interaction network diagram. The green nodes represent rice proteins, and the orange nodes represent M. oryzae proteins.
