# Supplementary figures and images for: Construction of sRNA Regulatory Network for Magnaporthe oryzae Infecting Rice Based on Multi-Omics Data
Source: Front Genet. 2021 Nov 12;12:763915. doi: 10.3389/fgene.2021.763915 (PMC8633311; doi:10.3389/fgene.2021.763915)

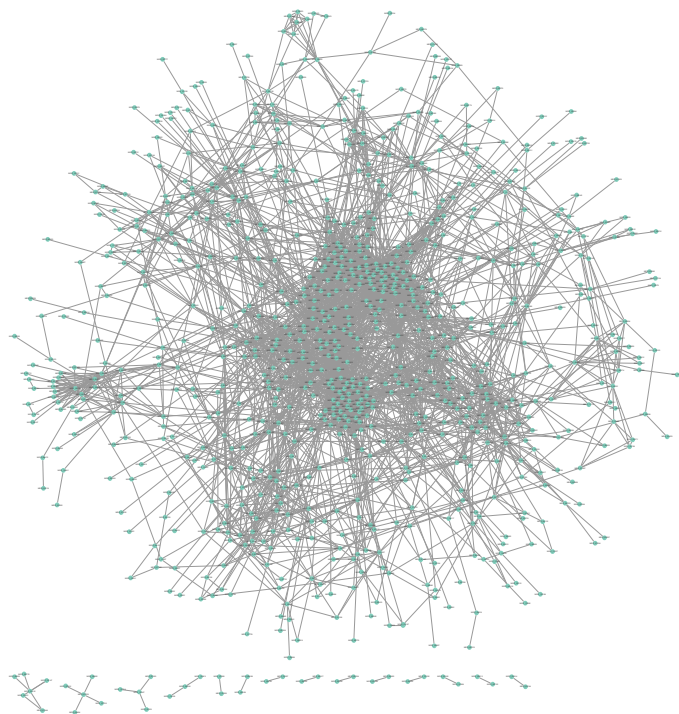

**Supplementary Figure 3.** PPI network diagram of *M. oryzae*.

Supplement: Supplementary file 11 [file Image3.PDF]
