# Supplementary material for: Construction of sRNA Regulatory Network for Magnaporthe oryzae Infecting Rice Based on Multi-Omics Data
Source: Front Genet. 2021 Nov 12;12:763915. doi: 10.3389/fgene.2021.763915 (PMC8633311; doi:10.3389/fgene.2021.763915)
Supplement: Supplementary file 12 [file Table2.DOCX]

**Supplementary Table 2.** GO function enrichment analysis table of rice core nodes.

| term description | fdr | gene name | subgroup |
| --- | --- | --- | --- |
| nucleosome | 2.69E-14 | H2B.10,H2B.7,H2B.6,H2B.5,H2B.11,OsJ_001872,H2B.9 | CC |
| protein-containing complex | 3.77E-13 | H2B.10,H2B.7,H2B.6,H2B.5,RPS27AA,H2B.11,OsJ_001872,OsJ_011822,OS05T0155601-00,H2B.9,NH5 | CC |
| nucleus | 7.94E-11 | H2B.10,H2B.7,H2B.6,H2B.5,OS01T0197200-01,RPS27AA,H2B.11,JMJ705,OsJ_001872,OS05T0155601-00,H2B.9,HOX5,NH5 | CC |
| intracellular non-membrane-bounded organelle | 8.52E-11 | H2B.10,H2B.7,H2B.6,H2B.5,OS01T0197200-01,RPS27AA,H2B.11,OsJ_001872,H2B.9 | CC |
| intracellular membrane-bounded organelle | 3.16E-10 | H2B.10,H2B.7,H2B.6,H2B.5,OS01T0197200-01,RPS27AA,H2B.11,JMJ705,FTSH7,OsJ_001872,OsJ_011822,OS05T0155601-00,H2B.9,HOX5,NH5 | CC |
| cell | 2.60E-08 | H2B.10,H2B.7,H2B.6,H2B.5,OS01T0197200-01,RPS27AA,H2B.11,JMJ705,FTSH7,OsJ_001872,OsJ_011822,OS05T0155601-00,H2B.9,HOX5,NH5 | CC |
| cytoplasm | 0.0098 | OS01T0197200-01,RPS27AA,FTSH7,OsJ_011822,OS05T0155601-00,NH5 | CC |
| nuclear lumen | 0.0016 | OS01T0197200-01,OsJ_001872,OS05T0155601-00 | CC |
| organelle envelope | 0.025 | FTSH7,OS05T0155601-00 | CC |
| protein heterodimerization activity | 2.81E-13 | H2B.10,H2B.7,H2B.6,H2B.5,H2B.11,OsJ_001872,H2B.9 | MF |
| binding | 5.52E-10 | H2B.10,H2B.7,H2B.6,H2B.5,OS01T0197200-01,CAM3,RPS27AA,H2B.11,JMJ705,FTSH7,AGO1C,OsJ_001872,OS05T0155601-00,H2B.9,HOX5,NH5 | MF |
| nucleic acid binding | 1.56E-09 | H2B.10,H2B.7,H2B.6,H2B.5,OS01T0197200-01,H2B.11,JMJ705,AGO1C,OsJ_001872,H2B.9,HOX5 | MF |
| protein binding | 1.52E-08 | H2B.10,H2B.7,H2B.6,H2B.5,H2B.11,OsJ_001872,H2B.9,NH5 | MF |
| organic cyclic compound binding | 4.33E-08 | H2B.10,H2B.7,H2B.6,H2B.5,OS01T0197200-01,H2B.11,JMJ705,FTSH7,AGO1C,OsJ_001872,H2B.9,HOX5 | MF |
| heterocyclic compound binding | 4.33E-08 | H2B.10,H2B.7,H2B.6,H2B.5,OS01T0197200-01,H2B.11,JMJ705,FTSH7,AGO1C,OsJ_001872,H2B.9,HOX5 | MF |
| structural molecule activity | 0.0113 | RPS27AA,OsJ_011822 | MF |
| DNA binding | 4.94E-07 | H2B.10,H2B.7,H2B.6,H2B.5,H2B.11,OsJ_001872,H2B.9,HOX5 | MF |
| negative regulation of gene expression | 0.0148 | OS01T0197200-01,AGO1C,OsJ_001872 | BP |
| regulation of macromolecule metabolic process | 0.0148 | OS01T0197200-01,JMJ705,AGO1C,OsJ_001872,HOX5,NH5 | BP |
| intracellular protein transport | 0.0226 | OsJ_011822,OS05T0155601-00 | BP |
| regulation of gene expression | 0.0226 | OS01T0197200-01,JMJ705,AGO1C,OsJ_001872,HOX5 | BP |
| regulation of gene expression, epigenetic | 0.0226 | JMJ705,OsJ_001872 | BP |
| gene silencing | 0.0228 | AGO1C,OsJ_001872 | BP |
| macromolecule metabolic process | 0.0242 | OS01T0197200-01,RPS27AA,JMJ705,FTSH7,HOX5,NH5 | BP |
| protein metabolic process | 0.0242 | RPS27AA,JMJ705,FTSH7,NH5 | BP |
| chromatin organization | 0.0274 | JMJ705,OsJ_001872 | BP |
| proteolysis | 0.0312 | FTSH7,NH5 | BP |
| nitrogen compound metabolic process | 0.0312 | OS01T0197200-01,RPS27AA,JMJ705,FTSH7,HOX5,NH5 | BP |
| cellular macromolecule catabolic process | 0.0312 | OS01T0197200-01,NH5 | BP |
| regulation of nitrogen compound metabolic process | 0.0312 | JMJ705,OsJ_001872,HOX5,NH5 | BP |
| regulation of primary metabolic process | 0.0312 | JMJ705,OsJ_001872,HOX5,NH5 | BP |
| cellular macromolecule metabolic process | 0.0359 | OS01T0197200-01,RPS27AA,JMJ705,HOX5,NH5 | BP |
| gene expression | 0.0332 | OS01T0197200-01,RPS27AA,JMJ705,HOX5 | BP |
| primary metabolic process | 0.0468 | OS01T0197200-01,RPS27AA,JMJ705,FTSH7,HOX5,NH5 | BP |
| cellular process | 0.0478 | OS01T0197200-01,RPS27AA,JMJ705,AGO1C,OsJ_001872,HOX5,NH5 | BP |
