# Supplementary material for: Construction of sRNA Regulatory Network for Magnaporthe oryzae Infecting Rice Based on Multi-Omics Data
Source: Front Genet. 2021 Nov 12;12:763915. doi: 10.3389/fgene.2021.763915 (PMC8633311; doi:10.3389/fgene.2021.763915)
Supplement: Supplementary file 13 [file Image11.PDF]

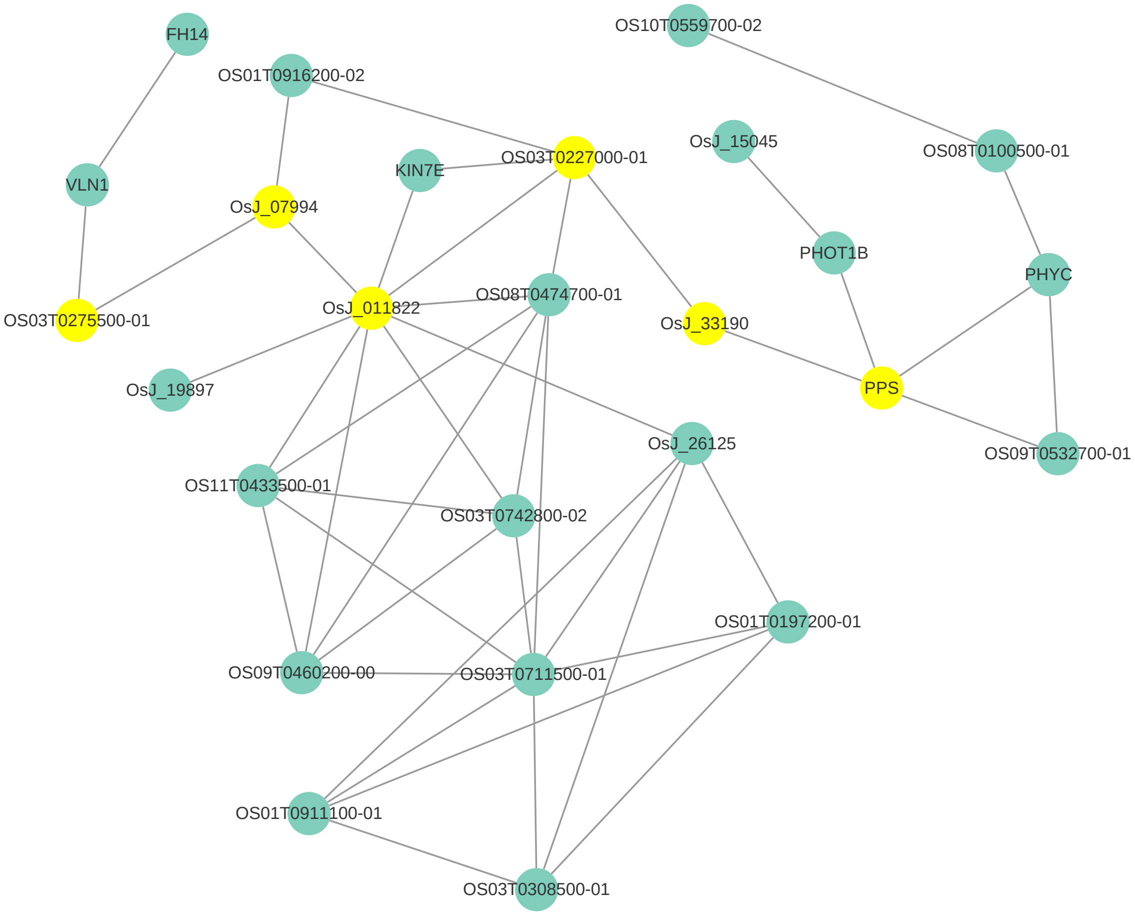

Supplementary Figure 11. Rice gene expression, transport and metabolism-related module (Cluster 8). Cluster 8 contains 26 gene nodes. In this section, the betweenness of each node is calculated according to the network topology attribute calculation method and sorted according to its criticality to nodes. The top 6 genes in betweeness ranking are selected as the central regulatory genes in Cluster 8, which are OS03T0227000-01, OsJ_011822, OsJ_33190, PPS, OsJ_07994, OS03T0275500-01, the genes with central regulatory function shown as yellow nodes in the network diagram.

This network module is mainly enriched in the biological process, cellular component and molecular function. One of the GO terms of the regulatory module is related to regulation of gene expression, such as regulation of gene expression, gene expression. The second is related to transport pathway of biomolecules, such as intracellular membrane-bounded organelle, vesicle-mediated transport, intracellular protein transport, etc. The third is related to rice metabolic pathways, such as RNA metabolic process, mRNA metabolic process, nitrogen compound metabolic process, primary metabolic process, cellular metabolic process, etc. These GO functional modules show that the infection process of M. oryzae affected the gene expression and metabolism of rice.
