# Supplementary material for: Construction of sRNA Regulatory Network for Magnaporthe oryzae Infecting Rice Based on Multi-Omics Data
Source: Front Genet. 2021 Nov 12;12:763915. doi: 10.3389/fgene.2021.763915 (PMC8633311; doi:10.3389/fgene.2021.763915)
Supplement: Supplementary file 14 [file Image7.PDF]

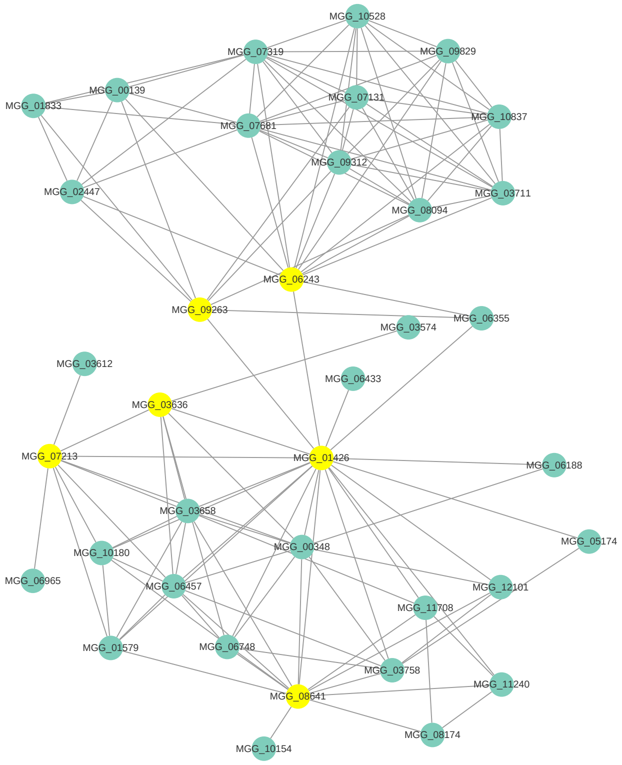

Supplementary Figure 7. M. oryzae gene expression and mRNA processing-related module (Cluster 4). Cluster 4 contains 37 gene nodes. In this section, the betweenness of each node is calculated according to the network topology attribute calculation method and sorted according to its criticality to nodes. The top 6 genes in betweeness ranking are selected as the central regulatory genes in Cluster 4, which are MGG_01426, MGG_06243, MGG_09263, MGG_ 07213, MGG_08641, MGG_03636, the genes with central regulatory function shown as yellow nodes in the network diagram.

A total of 11 apparently enriched GO functional items are involved in this subnet, and 8 related GO items are involved in RNA transcription, translation and protein synthesis. Among them, 3 GO items are involved in biological processes, namely GO:0006397, GO:0008380 and GO:0010468. There are two kinds of GO items related to molecular functions, namely GO: 0003676 and GO:0003723. There are 3 GO items related to cell components, namely GO: 0005681, GO:0032991 and GO:0005622.

There are 6 apparently enriched KEGG enrichment pathways in this subnet, and the KEGG enrichment pathways are arranged according to the P-value from small to large as follows. They are basic transcription factor enrichment pathway, RNA polymerase enrichment pathway, pyrimidine metabolism enrichment pathway, purine metabolism enrichment pathway, nucleotide excision repair enrichment pathway, ribosome biogenesis in eukaryotes enrichment pathway and metabolic pathway enrichment pathway.
