# Supplementary material for: Construction of sRNA Regulatory Network for Magnaporthe oryzae Infecting Rice Based on Multi-Omics Data
Source: Front Genet. 2021 Nov 12;12:763915. doi: 10.3389/fgene.2021.763915 (PMC8633311; doi:10.3389/fgene.2021.763915)
Supplement: Supplementary file 15 [file Image1.PDF]

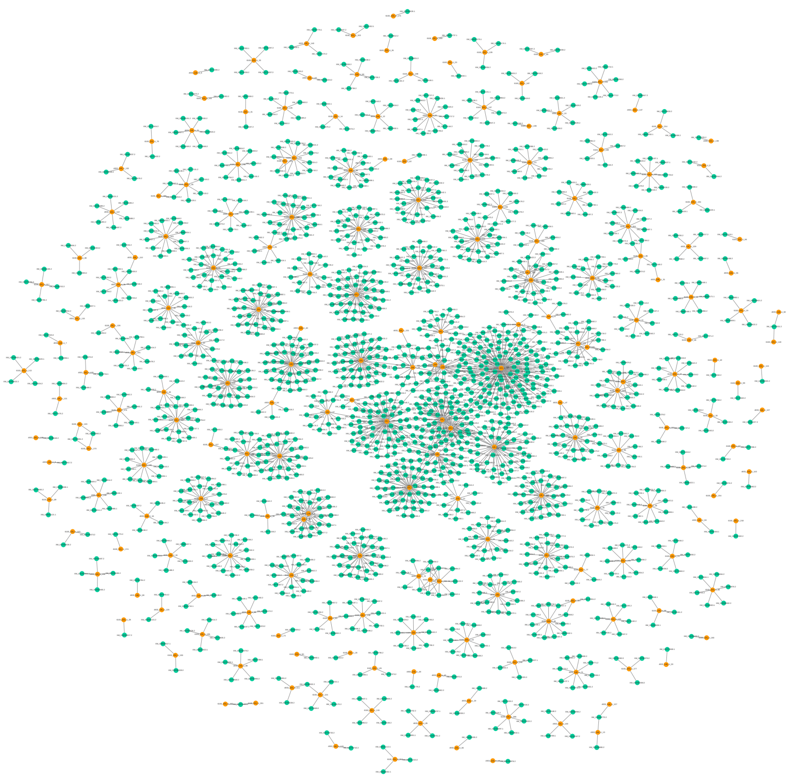

Supplementary Figure 1. Transcriptome network diagram of M. oryzae-rice. The green nodes are the mRNAs of rice, and the orange nodes are the sRNAs of M. oryzae.
