# Supplementary material for: Construction of sRNA Regulatory Network for Magnaporthe oryzae Infecting Rice Based on Multi-Omics Data
Source: Front Genet. 2021 Nov 12;12:763915. doi: 10.3389/fgene.2021.763915 (PMC8633311; doi:10.3389/fgene.2021.763915)
Supplement: Supplementary file 2 [file Image5.PDF]

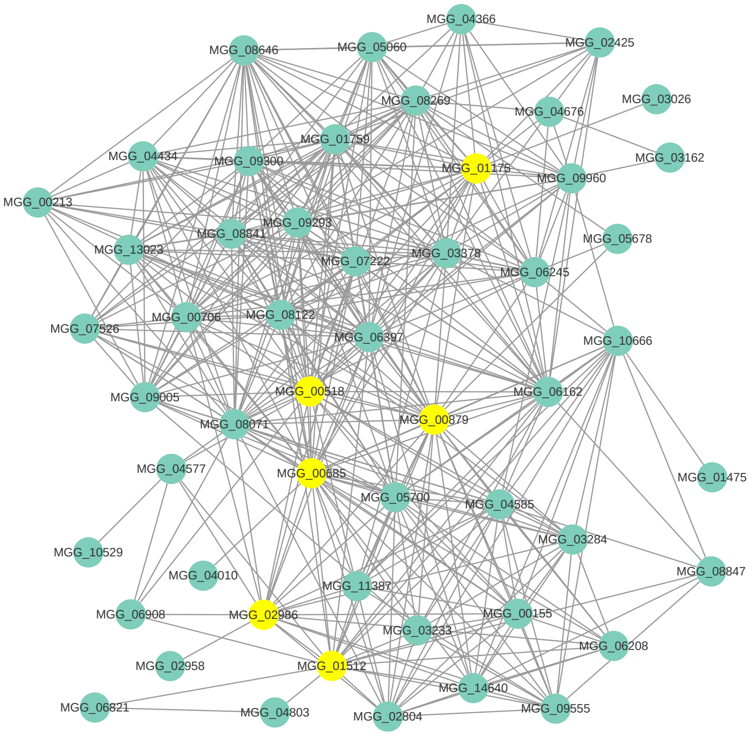

Supplementary Figure 5. M. oryzae DNA repair related module (Cluster 2). Cluster 2 contains 53 gene nodes. In this section, the betweenness of each node is calculated according to the network topology attribute calculation method and sorted according to its criticality to nodes. The top 6 genes in betweeness ranking are selected as the central regulatory genes in Cluster 2, which are MGG_01512, MGG_00685, MGG_00879, MGG_00518, MGG_02986, MGG_01175, the genes with central regulatory function shown as yellow nodes in the network diagram. Cluster 2 has no apparent enrichment GO pathway, so in this section, Cluster 2 is analyzed only through KEGG enrichment analysis. There are apparently enriched 11 KEGG enrichment pathways in Cluster 2, including nucleotide exicision repair pathway, homologous recombination and mismatch repair, etc. A total of 24 genes are involved, accounting for 53% of the total gene nodes.
